# B Cell Mobilization, Dissemination, Fine Tuning of Local Antigen Specificity and Isotype Selection in Asthma

**DOI:** 10.3389/fimmu.2021.702074

**Published:** 2021-10-14

**Authors:** Line Ohm-Laursen, Hailong Meng, Kenneth B. Hoehn, Nima Nouri, Yue Jiang, Chris Clouser, Timothy G. Johnstone, Ron Hause, Balraj S. Sandhar, Nadine E. G. Upton, Elfy B. Chevretton, Raj Lakhani, Chris J. Corrigan, Steven H. Kleinstein, Hannah J. Gould

**Affiliations:** ^1^ Randall Centre for Cell and Molecular Biophysics and School of Basic and Medical Biosciences, King’s College London, London, United Kingdom; ^2^ Asthma UK Centre in Allergic Mechanisms of Asthma, London, United Kingdom; ^3^ Department of Pathology, Yale School of Medicine, New Haven, CT, United States; ^4^ Center for Medical Informatics, Yale School of Medicine, New Haven, CT, United States; ^5^ Bristol Myers Squibb, Seattle, WA, United States; ^6^ Department of Ear, Nose and Throat (ENT) Services, Guy’s and St Thomas’ NHS Foundation Trust, London, United Kingdom; ^7^ Department of Respiratory Medicine and Allergy and School of Immunology and Microbial Sciences, King’s College London, London, United Kingdom; ^8^ Interdepartmental Program in Computational Biology and Bioinformatics, Yale University, New Haven, CT, United States; ^9^ Department of Immunobiology, Yale School of Medicine, New Haven, CT, United States

**Keywords:** asthma, immunoglobulin, allergy, adaptive immunity, IgD, innate immunity, repertoire and AIRR-seq/sequencing

## Abstract

In order to better understand how the immune system interacts with environmental triggers to produce organ-specific disease, we here address the hypothesis that B and plasma cells are free to migrate through the mucosal surfaces of the upper and lower respiratory tracts, and that their total antibody repertoire is modified in a common respiratory tract disease, in this case atopic asthma. Using Adaptive Immune Receptor Repertoire sequencing (AIRR-seq) we have catalogued the antibody repertoires of B cell clones retrieved near contemporaneously from multiple sites in the upper and lower respiratory tract mucosa of adult volunteers with atopic asthma and non-atopic controls and traced their migration. We show that the lower and upper respiratory tracts are immunologically connected, with trafficking of B cells directionally biased from the upper to the lower respiratory tract and points of selection when migrating from the nasal mucosa and into the bronchial mucosa. The repertoires are characterized by both IgD-only B cells and others undergoing class switch recombination, with restriction of the antibody repertoire distinct in asthmatics compared with controls. We conclude that B cells and plasma cells migrate freely throughout the respiratory tract and exhibit distinct antibody repertoires in health and disease.

## Introduction

The respiratory tract is located inside the body but, like the digestive and reproductive tracts, is constantly exposed to the external environment with its plethora of stimuli from microorganisms, proteins including allergens and other particulate matter including particulate pollution. The respiratory tract is divided into upper and lower regions above and below the larynx, the former comprising the nasal cavity, paranasal sinuses and pharynx and the latter the trachea and bronchial tree. It is also contiguous with the skin (*via* the nostrils) and the conjunctiva (*via* the nasolacrimal ducts). The respiratory tract mucosa serves as a physical barrier composed principally of pseudostratified cells, with ciliated columnar epithelial cells resting on a basement membrane along with mucin-producing goblet cells. The cellular composition of the lamina propria underneath the epithelial layer varies according to the site within the respiratory tract but includes blood vessels and lymphatics, structural cells and a range of immune cells including innate immune cells, antigen-presenting and effector cells, cartilage and, in the lower respiratory tract, smooth muscle cells. Lymphoid tissue surrounds the upper respiratory tract (Waldeyer’s Ring, which includes the lingual tonsils and the adenoids), while lymph nodes are abundant adjacent to the lower airways in the mediastinum and adjacent to the trachea and bronchi. Lymph flows towards the hilum and ultimately returns to the systemic venous circulation *via* the right lymphatic and left thoracic ducts.

In contrast to this prominent network of lymph nodes outside the lower airways, the question whether or not organized lymphoid tissue exists within the walls of normal airways (bronchial associated lymphoid tissue, or BALT), is still debated, although it is widely accepted that BALT can be “induced” (iBALT) in association with many chronic inflammatory diseases of the airways such as COPD (1–4). In addition, we have shown that the enzyme activation-induced cytidine deaminase (AID), which is necessary for both somatic hypermutation and isotype switching in B cells, is expressed in both healthy and asthmatic lung tissue (5). Both observations support the hypothesis that B cell maturation can take place within the walls of the airways themselves.

While the process of immune cellular migration from the blood vessel capillaries into the tissues and their return to the systemic circulation *via* the lymph nodes and lymphatics is relatively well established, little is known about the capacity of *individual* immune cells such as B cells and plasma cells to migrate freely between the mucosal surfaces and lymphatics of the upper and lower respiratory tracts. According to the concept of the “United Airways Disease” (UAD) treatment of disease at one site in the respiratory tract, such as allergic rhinitis, will result in improvement in symptoms in another, such as the lower respiratory tract in allergic asthma (6). This is consistent with the hypothesis that such B cell migration within the respiratory tract may occur.

We previously characterized the expressed antibody repertoires of the bronchial mucosal B cells at various sites within the lower respiratory tract, obtained at bronchial biopsy, and the peripheral blood in one patient with atopic asthma (AA) and one non-atopic, non-asthmatic (NANA) control subject using Adaptive Immune Receptor Repertoire sequencing (AIRR-seq). Detailed analysis of the sequences provided conclusive evidence that B cells are able to migrate both locally and to more distal sites within the bronchial mucosa of the same lung (7). The study also, for the first time, revealed the presence of so-called IgD-only cells in the bronchial mucosa, i.e. B cells expressing surface IgD but not IgM generated through isotype switching utilizing a cryptic switch region upstream of the ∂ constant region (8, 9). These cells were first described as highly mutated germinal center centroblasts (10). It has been suggested that this switching process is driven by bacteria (8), which could include common commensal bacteria in asthma, making the discovery of bronchial IgD-only cells relevant to our aim to better understand asthma pathogenesis.

In the present study a primary goal was to extend these observations by addressing the hypothesis that B cells in the upper (nasal) and lower (bronchial) respiratory tract mucosa of both lungs are directly related. We compared the repertoires of the B cells obtained near contemporaneously from nasal biopsies (NBx) and bronchial mucosal biopsies (BBx) (including biopsies from both the left and right bronchial trees from one individual) and the peripheral blood from patients with atopic asthma (AA) and non-atopic, non-asthmatic control subjects (NANA). Our secondary aim, through detailed analysis of somatic hypermutation of the B cell antibody genes, was to attempt to align their directions of travel with their maturation history. Thirdly and finally, we wished to confirm and extend our previous observation of IgD-only cells in the bronchial mucosa in a larger cohort and to compare IgD-only cells of the nasal and bronchial mucosa.

## Materials and Method

### Study Subjects and Sample Collection

**Figure 1:**
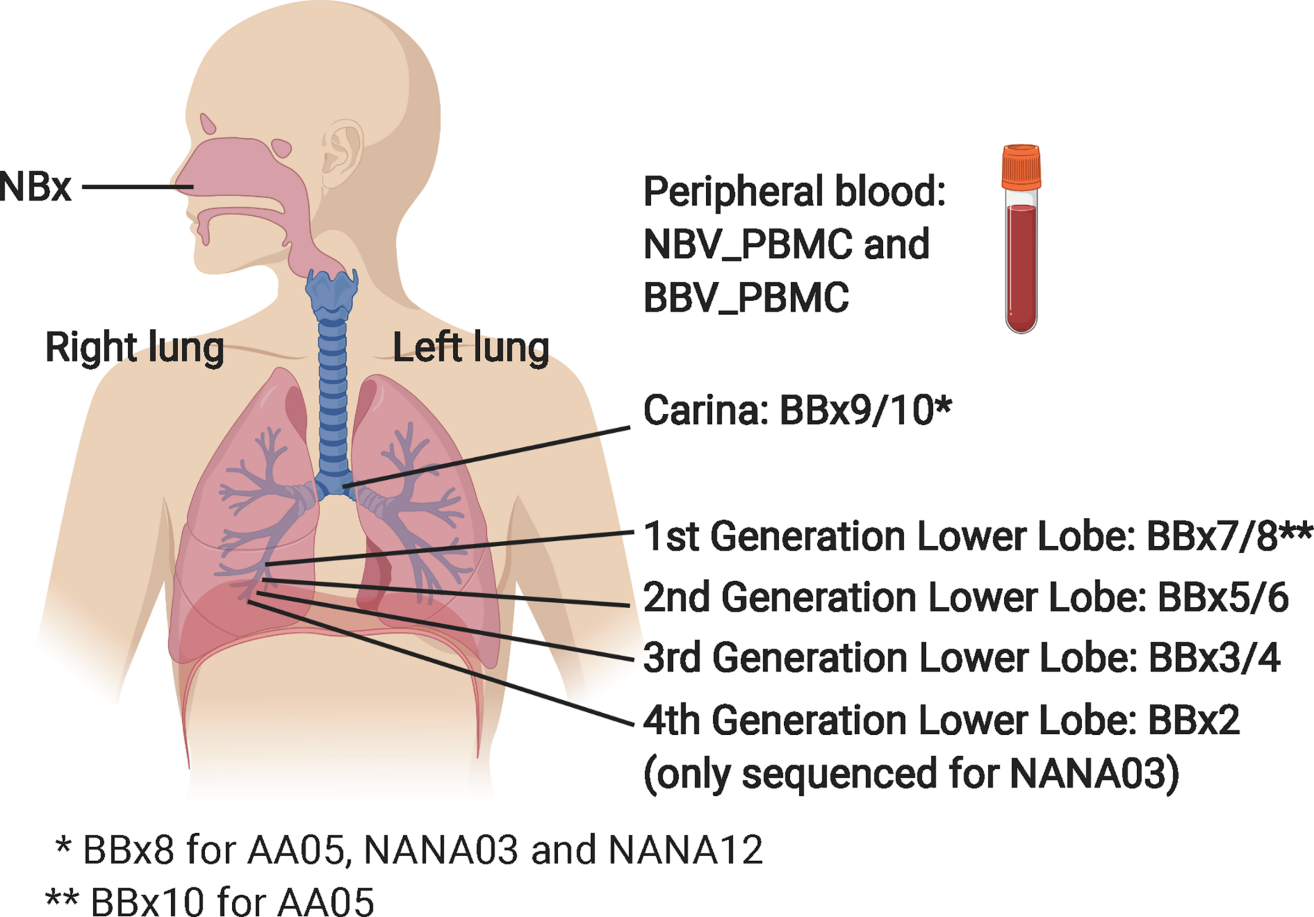
Schematic showing origins of the various samples from the nasal mucosa, the right lung bronchial mucosa and the peripheral blood. The NBV_PBMC and BBC_PBMC peripheral blood sample were collected at the same time as the nasal biopsy (NBx) and the bronchial biopsies (BBx), respectively. For AA07, three biopsies from the left lung were also collected: BBx11 (3^rd^ generation lower lobe), BBx12 (2^nd^ generation lower lobe) and BBx13 (1^st^ generation lower lobe). The carina is at the bottom of the trachea where it divides into the left and right main bronchi. 4^th^ generation lower lobe biopsies were collected from all subjects but only sequenced from NANA03 as this individual lacked a 1^st^ generation lower lobe biopsy sample. This figure was created in BioRender.com.

Six atopic patients with asthma (AAXX) and nine non-atopic, non-asthmatic controls (NANAXX) were enrolled in the study (**Supplementary Table 1**). The criteria for the diagnosis of asthma and atopy have been described previously (11). From each subject, two biopsies of the nasal mucosa were obtained from the inferior turbinates at anterior rhinoscopy under local anaesthesia and, at a second visit 4-43 days later, 10 bronchial mucosal biopsies from various sites in the lower airways at fibreoptic bronchoscopy under local anaesthesia (**Figure 1**). For subject AA07, 13 bronchial biopsies were collected, as we also obtained three biopsies from the lower lobes of the right lung of this subject. At both visits 30 ml peripheral blood were also collected for PBMC and serum isolation.

### RNA Extraction, AbSeq Library Preparation and Sequencing

Total RNA was extracted from one of the paired nasal biopsies and one bronchial biopsy from each of four sites within the bronchial mucosa using a Qiagen RNEasy plus kit. One aliquot of 10^6^ PBMCs from each blood sample was carefully thawed and RNA extracted using the Promega Maxwell HT simply RNA custom kit. RNA recovery and quality were estimated using Agilent TapeStation and HSRNA ScreenTapes.

The RNA was used for heavy chain immunoglobulin amplification and high-throughput sequencing using the previously described AbSeq protocol (12). Briefly, RNA was reverse-transcribed into cDNA using a biotinylated oligo dT primer. An adaptor sequence was added to the 5’ end of all cDNA, which contains the Illumina P7 universal priming site and a 17-nucleotide unique molecular identifier (UMI). Products were purified using streptavidin-coated magnetic beads followed by a primary PCR reaction using a pool of primers targeting the IGHA, IGHD, IGHE, IGHG, and IGHM regions, as well as a sample-indexed Illumina P7C7 primer. The immunoglobulin-specific primers contained tails corresponding to the Illumina P5 sequence. PCR products were then purified using AMPure XP beads. A secondary PCR was then performed to add the Illumina C5 clustering sequence to the end of the molecule containing the constant region. The number of secondary PCR cycles was tailored to each sample to avoid entering the plateau phase, as judged by a prior quantitative PCR analysis. Final products were purified, quantified with Agilent Tapestation and pooled in equimolar proportions, followed by high-throughput paired-end sequencing on the Illumina MiSeq platform. For sequencing, the Illumina 600 cycle kit was used with modifications: 325 cycles were used for read 1, 6 cycles for the index reads, 300 cycles for read 2 and a 20% PhiX spike-in to increase sequence diversity.

### AbSeq Sequence Analysis

Briefly, data were processed and analyzed using the Immcantation Framework (http://immcantation.org) with the Presto v0.5.7 (13), Change-O v0.4.0 (14), Alakazam v0.2.10 (14), SHazaM v0.1.9 (14, 15), Dowser v0.0.1 (16), IgPhyML v1.1.3 (17) and other custom scripts through the R environment (18). Filtering steps included discarding sequences aligned against the PhiX174 reference genome, sequences with mismatches in the constant region, sequences with more than six mismatches in a 10 nucleotide stretch and sequences with only one single contributing read. Non-functional sequences were also removed from the data, and clonally related sequences were identified using a clustering-based approach as detailed in the **Supplementary Methods**. A detailed description of the various analyses performed can be found in figure legends and **Supplementary Methods**.

### AbPair Single Cell Sequencing

From five selected asthmatics and four non-atopic controls (marked in **Table 1**) we also prepared PBMCs to sequence the paired heavy and light chain sequences using the AbPair protocol (19). Selection was based on the finding of shared IgD and/or IgE clones between tissue samples (nasal and/or bronchial mucosa) and a PBMC sample in the data generated by AbSeq. In the analysis of AbPair sequences, the heavy chain sequences from single cell data were extracted and combined with bulk data and then analyzed using the Immcantation pipeline. A detailed description can be found in **Supplementary Methods**.

**Table 1:** Numbers of sequences and clones from the various individuals and samples.

Identifier	Days btw. samples	NBx*No. of sequences(No. of clones)	NBV_PBMC*No. of sequences(No. of clones)	BBx*No. of sequences(No. of clones)	BBV_PBMC*No. of sequences(No. of clones)
**Atopic Asthmatics (AA)**					
AA02	28	19933 (8190)	7100** (4502)	8013 (1534)	14756** (8344)
AA03	23	15508 (5943)	693 (191)	4092 (1638)	5765** (3516)
AA04	32	17229 (7936)	426 (113)	13646 (4356)	1489 (962)
AA05	6	19717 (8877)	1401** (768)	4126 (1487)	386 (108)
AA06	21	13623 (6891)	3916 (1898)	18852 (6119)	626 (293)
AA07	4	13364 (6047)	2570** (1859)	21939*** (5073)	19 (9)
**Non-Atopic, Non-Asthmatic controls (NANA)**					
NANA01	38	13115 (5055)	28976** (21016)	19398 (4807)	14636** (9257)
NANA03	29	27556 (10539)	640 (528)	8809 (3297)	1618 (859)
NANA04	7	35805 (10951)	4 (4)	17034 (5517)	8736** (7060)
NANA05	33	41013 (11082)	33877 (26559)	16957 (5491)	2973 (2026)
NANA07	26	41549 (12165)	17250 (2366)	18994 (6141)	4532 (1595)
NANA08	29	16333 (8246)	2556** (2086)	22065 (7645)	1385 (518)
NANA10	43	14037 (6249)	582 (401)	10082 (3748)	713 (282)
NANA11	13	12976 (5451)	571 (215)	14482 (3979)	410 (99)
NANA12	18	11009 (5041)	6467 (3272)	13485 (4909)	3885 (1924)

*NBx, nasal mucosal biopsy; NBV_PBMC, peripheral blood cells from a blood sample taken at the same time as the nasal biopsy; BBx, bronchial musical biopsy and BBV_PBMC, peripheral blood cells from a blood sample taken at the time of the bronchoscopy.

**AbSeq and AbPair data combined.

***This number includes 958 sequences from the carina, 11093 sequences from biopsies from the right lung mucosa and 9888 sequences from biopsies form the left lung mucosa.

### Statistical Analyses

Unless specifically stated, all statistical analyses throughout the study were performed as two-tailed Wilcoxon rank sum tests to test whether or not samples from two groups were likely to originate from the same population.

### Study Approval

All 15 study participants provided written, informed consent to take part in the study in accordance with the Declaration of Helsinki. The study had been approved by a local research ethics committee (REC number 15/LO/1800).

## Results

### Almost 700,000 High-Quality B Cell Receptors From Nasal Biopsies, Bronchial Biopsies and Peripheral Blood B Cells From 15 Individuals Were Sequenced and Analyzed

We enrolled six atopic asthmatics (AA) and nine non-atopic, non-asthmatic control subjects (NANA) into the study (see **Supplementary Table 1**). From each we collected nasal biopsies (NBx), bronchial biopsies (BBx) from multiple sites within the lower airways including the carina and peripheral blood (**Figure 1**). Total RNA was extracted from one of the paired nasal biopsies, one bronchial biopsy from each of the four bronchial sites and an aliquot of PBMCs from each of two blood samples (NBV_PBMC taken at the time of the nasal biopsy and BBV_PBMC taken at the time of the bronchoscopy). The RNA was used for heavy chain immunoglobulin amplification and sequencing using the previously described AbSeq protocol (12). A total of 48,731,069 heavy chain sequences were generated. After multiple steps of quality control (QC), filtering and trimming (see Materials and Methods), a total of 693,700 unique high-quality sequences, ranging from 25,414 to 94,820 per subject, were retained and used for analyses (see **Table 1** for a breakdown). Rarefaction analysis (**Supplementary Figure 1**) showed that, although we had not saturated the diversity for any sample type, the coverage was generally good.

### High Degree of Overlap Between the B Cell Repertoires of the Left and Right Lung and Carina Bronchial Mucosa

**Figure 2:**
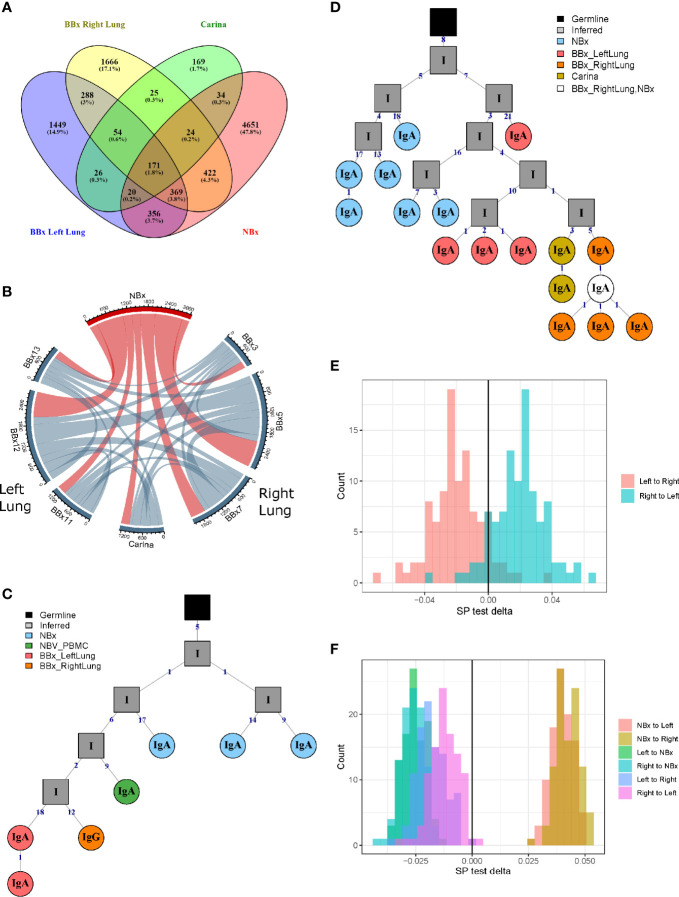
B cells can traffic between the left and the right lungs, as well as from the carina and the nose to the lower airways. **(A)** Venn diagram showing the numbers (and percentages) of clones that overlap in the left and the right lungs, carina and the nose from the asthmatic patient AA07. **(B)** Clonal relationships between all seven bronchial biopsies (BBx) and the nasal biopsy (NBx) from AA07 were calculated and plotted in a Circos plot. The lines between regions indicate a clonal relationship and the numbers around the perimeter indicate counts of overlapped clones between two biopsies. Clones that overlapped more than two biopsies could be duplicated in the Circos plot. **(C, D)** Examples of clonal trees for two clones from AA07 spanning both lungs (right: orange and left: red), the nose (blue) and, for the clones in **(C)**, also the peripheral blood (green). The clone in **(D)** also has members from the carina (yellow). The clone in **(C)** has undergone isotype switching and contains both IgA and IgG sequences. The numbers along each of the branches indicate mutations. **(E)** Maximum parsimony phylogeography analyses show no enrichment for trafficking between the mucosa of the two lungs in either direction. **(F)** When the nasal clones are included, the analyses reveal enrichment for trafficking from the nasal mucosa to either lung (peach and yellow) whereas trafficking between the mucosa of the two lungs (purple and pink) shows no directional bias. There is negative enrichment for travel from either lung to the nasal mucosa (green and blue) confirming the directional preference for B cell trafficking from the nasal to the bronchial mucosa.

From one of the atopic asthmatic subjects, AA07, we obtained near contemporaneous biopsies from the bronchial mucosa of the right and left lungs, allowing detailed analysis of the clonal relationships between B cells at the two sites (see **Supplementary Materials and Methods**). We found a high degree of B cell clonal overlap in the left and right bronchial mucosa (38 and 29%, respectively), and many of these clones were also shared with the carina and/or the nose (**Figure 2A**). If a B cell clone was shared between the bronchial mucosa of both lungs and the carina, it was more likely also to have members in the nasal mucosa (171 clones shared between the four sites *versus* 54 only found in the three lung sites). These expanded clones were often large (up to 512 unique sequences) compared to the local clones found in only one site (maximum of 28 unique sequences in the nasal and 58 in the bronchial biopsies). Connectivity analysis (**Figure 2B**) revealed the potential for clonal overlap between all of the sampled sites in the airway mucosa, confirming that the sites are immunologically connected and that no specific connections seem to be favored. Phylogenetic reconstruction of the pattern of accumulated somatic hypermutation (SHM) (**Figures 2C, D**) suggested that individual clones expand and disperse to occupy the bronchial mucosa of both lungs, the carina, nasal mucosa and/or peripheral blood. Isotype switching from IgA to IgG along the pathway of migration was also apparent in some of the lineages (example in **Figure 2C**).

To determine whether the connectivity of B cell clones in the left and right bronchial mucosa were different in this individual (AA07), we analyzed the pattern of trafficking events between tissues along B cell lineage trees (see Materials and Methods). Briefly, given the tissue (e.g., nasal, left or right lung bronchial mucosa) associated with each observed sequence in a lineage tree, the algorithm determines the set of internal node tissue states requiring the fewest number of trafficking events along the tree. The types of trafficking events (e.g., trafficking from left to right lung) that occur within each tree are then compared to those obtained from the same tree topologies with randomized tissue states (16). We first considered only sequences from the left or right lung mucosa and found no significant bias in the directions of trafficking between the two lungs (**Figure 2E**). Next, considering sequences from the nasal, left or right lung mucosa together, we observed a significantly greater proportion of trafficking events from the nasal mucosa to the bronchial mucosa of either lung (p<0.01 for both, **Figure 2F** and **Supplemental Table 2**). We also observed fewer total transitions in B cell lineage trees (mean=2949.3) compared to randomized trees (mean=3664.1) in all 100 simulation repetitions (p<0.01), showing that sequences from the same tissue were more similar than expected by chance. These results indicated a biased ancestor/descendent relationship within these trees from the nasal mucosa to the bronchial mucosa of both lungs, but not from the left or right bronchial mucosa to the other. This is consistent with biased B cell trafficking from the nose to the lungs, with no evidence for biased trafficking between the mucosa of the two lungs. A number of B cell clones spanning these tissues also had members in the peripheral blood (see example phylogenetic tree in **Figure 2C**) suggesting that this trafficking could take place, at least partially, *via* the circulation.

The clonal sequences confined to the left or right bronchial tree or carina shared similar characteristics in terms of mutation frequency, expansion, isotype distribution and numbers of isotypes within a clone (**Supplementary Figures 2A–D**, respectively). Thus, sampling either lung can provide B cell repertoire data representative of the entire bronchial mucosa. In the rest of the subject cohort (five atopic asthmatics and nine non-atopic controls in total), we sampled only the right lung.

### Differences in Clonal and Antigen-Driven Diversity in the Asthmatic Respiratory Tract Mucosa Compared to Non-Atopic Controls

**Figure 3:**
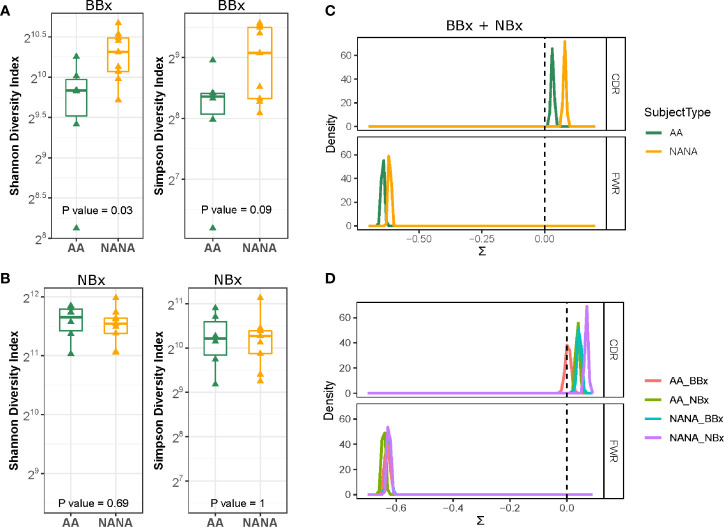
Greater diversity and positive immunoglobulin selection pressure in non-atopic, non-asthmatics (NANA) compared with atopic asthmatics (AA). The Shannon diversity and Simpson diversity indices of immunoglobulin sequences from biopsies from each individual show significantly less diversity in the asthmatics (green) compared with the non-atopic controls (yellow) in the bronchial **(A)** but not the nasal biopsies **(B)**. Comparison of **(C)** pooled bronchial and nasal biopsy immunoglobulin sequences from asthmatics and non-atopic control samples (green and yellow, respectively) and **(D)** bronchial (red) and nasal (green) biopsy sequences from the atopic asthmatics and bronchial (blue) and nasal (purple) sequences from the non-atopic controls shows a higher degree of antigen-driven selection in the complementarity determining regions (CDRs) of the antibodies in the non-atopic controls compared with the atopic asthmatics. Statistically significant differences between the subject groups were found in the framework regions (FWR) when bronchial and nasal biopsy samples were combined **(C)** but not for individual sample types **(D)**.

Trafficking is a feature of ongoing humoral immune responses often also characterized by clonal expansion of B cells, which can lead to loss of clonal diversity (20). We used the method of Hill (21) to measure various aspects of diversity and to compare diversity in the mucosa of the upper (nose) and lower (bronchi) respiratory tract and between asthmatics and control subjects (**Supplementary Figure 3**). The Hill plots were made using re-sampling to correct for varying sequencing depth of the analyzed populations and indicated that the diversity in sequences from the bronchial mucosa was more restricted in the asthmatics compared to the non-atopic controls, as confirmed by a significantly lower Shannon diversity index (p=0.03) (**Figure 3A**). We observed a similar trend for the Simpson diversity index, but this did not reach statistical significance. No differences in diversity between the groups were observed in the nasal mucosa (**Figure 3B**). Overall, the bronchial, but not the nasal mucosa, of the asthmatic subjects contained fewer unique sequences with greater clonal expansion suggesting an ongoing immune response perhaps with a more restricted number of driving antigens.

BASELINe analyses confirmed a positive selection pressure in the complementarity determining regions (CDRs) and a negative selection pressure in the framework regions (FWR) consistent with antigen-driven selection in both asthmatics and control subjects (**Figure 3C**). Interestingly, the analyses showed statistically significantly lower selection strength scores (∑) for both the CDRs and FWRs of the tissue samples from atopic asthmatics compared to the non-atopic controls (p=2.3^-9^ and 0.0004, respectively). Estimating the selection strength separately for nasal and bronchial biopsy samples, we still found statistically lower selection pressure scores in the CDRs of asthmatics compared to controls (p<0.0005 for both bronchial and nasal samples) (**Figure 3D**), but no differences were seen in the FWRs. We also found that the selection pressure was significantly higher in the nasal biopsies compared to the bronchial biopsies within both groups (atopic asthmatics and non-atopic controls) (p<0.005). This confirms that the antigenic selection pressure that the sequences from the bronchial and nasal tissue have encountered is different and also that sequences from atopic asthmatics and non-atopic controls have been exposed to different selection pressure.

### Both the Nasal and Bronchial Mucosa B Cell Repertoires Are Dominated by Mutated, Isotype Switched Immunoglobulin Sequences Utilizing a Broad Range of IGHV-Genes

**Figure 4:**
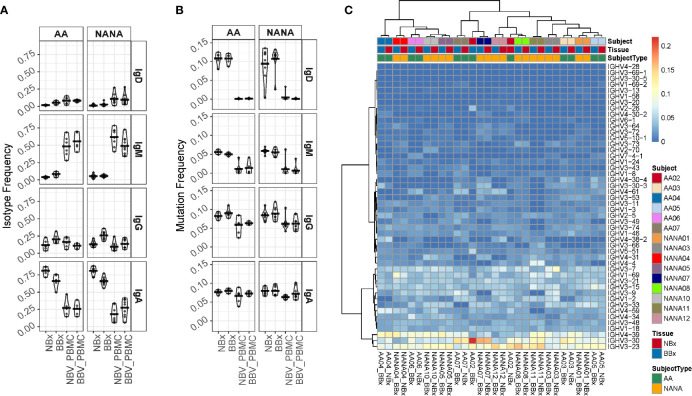
Analysis of isotype frequencies, levels of somatic hypermutation (SHM) and IGHV-gene usage of Ig clones from the bronchial (BBx) and nasal mucosal biopsies (NBx) and peripheral blood mononuclear cells of the atopic asthmatics and non-atopic, controls. **(A)** Isotype frequencies in samples were calculated as fractions of the total sequences expressing a given isotype. Each dot represents an individual. **(B)** SHM frequencies were calculated for each sequence, then means calculated for all the clones of a given isotype and then for each isotype in each tissue type for each individual. Each dot represents an individual. **(C)** Heatmap showing the IGHV-gene usage frequencies in the bronchial and nasal mucosal biopsies from each individual.

Diversity in antigen specificity and effector function is caused by the recombination of a variety of different IGHV-genes and constant region gene (IGHC), respectively. We first examined the isotype usage and found that in all subjects, atopic asthmatics as well as non-atopic controls, the nasal mucosa was dominated by IgA (70-90%, mean 85.9% of unique sequences) followed by IgG (mean 10.3% of unique sequences), but also included small amounts of IgM (mean 2.4% of unique sequences) and IgD (mean 1.4% of unique sequences) (**Figure 4A** and **Supplementary Figure 4** for individual samples). In the bronchial mucosa, IgA was still the dominant isotype in most samples (30-80%, mean 67.8% of unique sequences) although the levels were significantly lower than in the nasal mucosa (p<0.05). We found more IgG (up to 50%, mean 24.1% at sequence level), more IgM (4.2% of unique sequences) and significantly more IgD (3.9% of unique sequences, p<0.05) compared to the nasal mucosal samples. This was in contrast to the peripheral blood, where samples were dominated by IgM (30-70% of unique sequences), which was significantly increased compared to the tissue samples (p<0.05 for all comparisons). Owing to technical issues with the IgE-specific primers, we identified only small numbers of IgE sequences, which were insufficient for further, detailed analysis. In the lower airways, there were no significant isotype distribution differences between the asthmatic and non-atopic control subjects (p>0.05 for all comparisons), but we found it noteworthy that more IgD sequences were present in the lower airways from the asthma patients compared to the non-atopic controls. 99% of these IgD sequences were part of clones that did not include IgM sequences, referred to as IgD-only. We previously reported, for the first time, a high incidence of these IgD-only clones in the bronchial mucosa of a single asthmatic patient (7).

Comparing the degree of SHM in the B cell clones from the asthmatics and non-atopic controls, we found no significant differences in any compartment (tissue or peripheral blood) (**Figure 4B**). However, we found that the clones from both tissues carried significantly more mutations than their isotype-matched blood counterparts (p<0.05 for all comparisons) with the exception of IgA from nasal biopsies of asthmatics and IgA from bronchial biopsies of controls, which both exhibited the same trend but were not statistically significant (p=0.078 and 0.082, respectively). For IgD the mean SHM frequency was 10-11% in tissue but mostly un-mutated in blood and for IgM mean SHM frequency was 5-6% in tissue and 1% in blood. While these differences might in theory be explained by greater numbers of naïve B cells in the peripheral blood compared to tissue, we also found a difference for IgA (mean SHM 8% in tissue and 6-7% in blood) and IgG (mean SHM 8-9% in tissue and 6% in blood). Thus, there is clear evidence of selection of more somatically mutated clones in the tissues compared to the peripheral blood.

B cells in all samples, tissue as well as blood, utilized a broad spectrum of IGHV-genes (**Figure 4C** and **Supplementary Figure 5**) with IGHV3-23, IGHV3-30 and IGHV4-39, being the most commonly used in all subsets, which is in line with a previous report (22). Each individual had a unique IGHV-gene usage profile as evidenced by the fact that the nasal and bronchial mucosal samples from the same individual clustered with each other in all but one individual (AA02) (**Figure 4C**). No consistent differences were found between the bronchial and nasal samples or between the two subject groups suggesting that the overall IGHV-gene usage is not significantly affected by disease status. A few genes were statistically significantly more (IGHV1-18 and IGHV1-3) or less (IGHV3-23 and IGHV4-34) utilized when comparing tissue with blood samples (FDR<0.05) (data not shown) and the overall pattern of IGHV-gene usage in tissue and peripheral blood differed.

### Directional B Cell Trafficking From the Nasal to the Bronchial Mucosa

**Figure 5:**
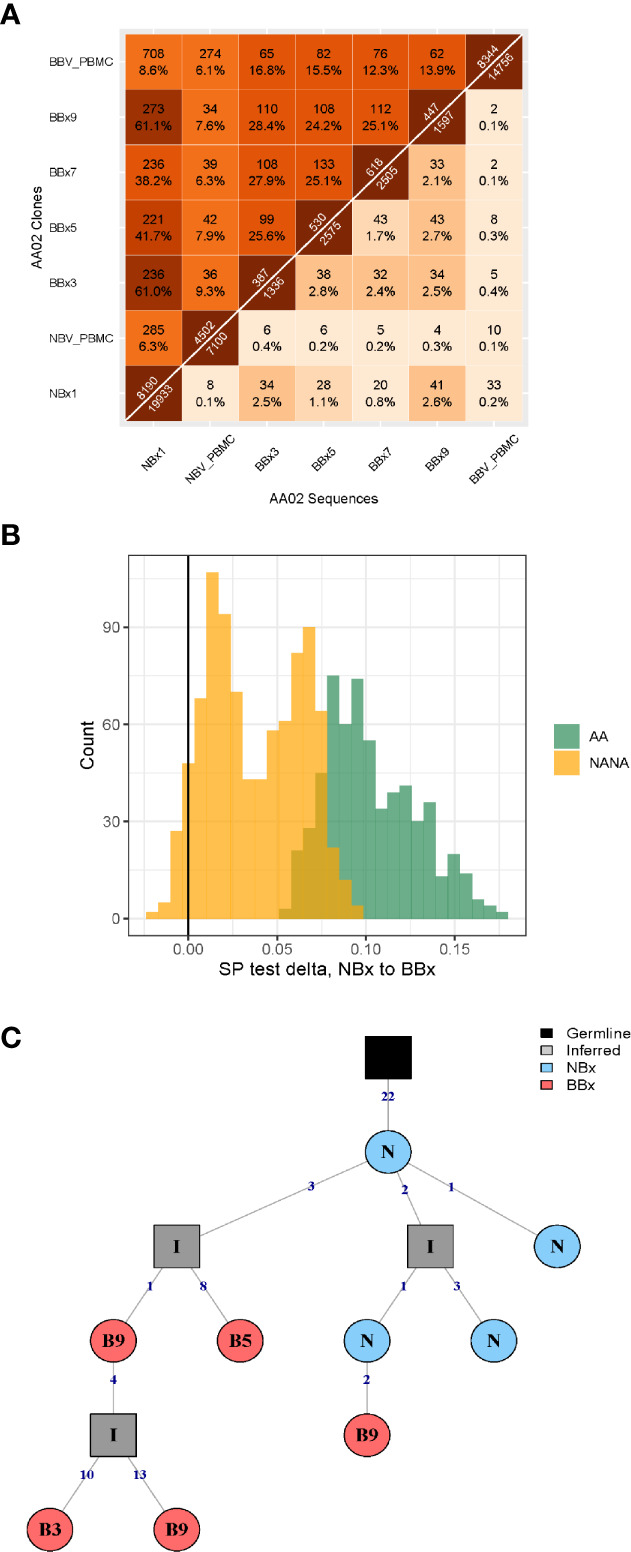
High frequency of immunoglobulin gene clones spanning nasal and bronchial mucosa and evidence of enrichment of trafficking from the nose to the lungs. **(A)** Overlap heatmap showing the numbers and frequencies of sequences (below the diagonal) and clones (above the diagonal) shared between all pairs of samples from subject AA02. Numbers immediately above and below the diagonal indicate the number of sequences and clones in each sample, respectively. There is a high degree of overlap of clones in the nasal biopsy (NBx1) and all the bronchial biopsies (BBx) (38-61% at clonal level) and between individual bronchial biopsies (24-28% at clonal level). Some overlap of clones is also seen in the two blood samples taken at the same times as the nasal and bronchial biopsies (6% at clonal level) as well as between both samples and the nasal biopsy (6% and 9%, respectively) and bronchial biopsy samples (6-9% for the NBV_PBMC taken at the time of the nasal biopsy and 12-19% for BBV_PBMC taken at the time of the bronchoscopy). A lower degree of overlap is found at sequence level, as this requires 100% nucleotide sequence identity. **(B)** Lineage tree analysis by maximum parsimony phylogeography showing an enrichment of switches along B cell lineage trees from nasal to bronchial mucosa compared to randomized trees. The enrichment is significantly more pronounced in the atopic asthmatics (AA, green, p<0.02 for all subjects) compared to the non-atopic controls (NANA, yellow, p<0.05 in 4 of 9 subjects). **(C)** Example of a phylogenetic tree from subject AA06 showing a clear pattern of trafficking from the nose (N, blue) to the lungs (bronchial biopsies B3, B5 and B9, red). N is an abbreviation for nasal biopsy (NBx) and B3, B5 and B9 abbreviations of bronchial biopsies BBx3, BBx5 and BBx9. All the members of this particular clone are of the IgA isotype.

We have previously shown that B cells can traffic within the local lung mucosa as well as to more distal sites within the mucosa of a single lung (7) and between lungs (here). To extend our previous study, we here included the nasal mucosa as a more distant site within the respiratory tract. Overlap plots showed a high degree of clone sharing between all individual bronchial biopsies and the nasal biopsy but to a lesser degree with the two blood samples (**Figure 5A**). Clones spanning both the nasal and the bronchial mucosa tended to have more mutations in the bronchial members than the nasal clone members, which attained statistical significance in all 6 of the asthmatic subjects and in 4 of the 9 non-atopic controls (see **Supplementary Table 3**).

We further investigated the direction of B cell trafficking between the nasal and bronchial mucosa of all subjects using B cell lineage trees and the approach described in Materials and Methods. Considering only sequences from nasal and bronchial biopsies, we identified fewer total trafficking events along the B cell lineage trees compared to trees with randomized tissue states at the tips in all individuals (p<0.01 for each). This indicates that sequences from the same tissue (nasal or bronchial mucosa) were more similar than would be expected by chance. Using the same method, we further identified a significantly greater proportion of trafficking events from nasal to bronchial mucosa than in randomized trees in 6/6 atopic asthmatics, and in 7/9 non-atopic control individuals (p<0.05 for each; **Supplementary Table 4**). These results confirmed a biased ancestor/descendent relationship from the nasal mucosa to the bronchial mucosa within these trees that was more evident in asthmatics (**Figure 5B**). We found no correlation between the strength of this relationship and either the proportion of sequences from nasal biopsies or the elapsed time between biopsies (**Supplementary Figures 6A, B**, respectively). This confirmed that these results were unlikely to result from either biased sampling (16) or accumulation of additional mutations between biopsies. When analyzing sequences from all tissue samples together (nasal and bronchial mucosa and peripheral blood at two time points), we found a significantly greater proportion of trafficking events from the nasal to the bronchial mucosa in 14/15 individuals (p<0.05 for each; **Supplementary Table 5**). All other directional comparisons only showed a significantly greater proportion of trafficking events in a maximum of two individuals suggesting no general importance. Overall, these results indicate that a biased ancestor/descendant relationship from the nasal to the bronchial mucosa is the dominant pattern in the analyzed trees (*e.g.* in **Figure 5C**), providing further evidence that, particularly in asthmatics, the immune response can be initiated in the nose and that activated B cells can then traffic to the bronchial tissue, accumulating mutations either within the bronchial mucosa or at unknown sites *en route* from the nasal to the bronchial mucosa.

### Trafficking of B Cell Clones of All Isotypes Between the Nasal and Bronchial Mucosa Is Linked to Isotype Switching and/or Selection

**Figure 6:**
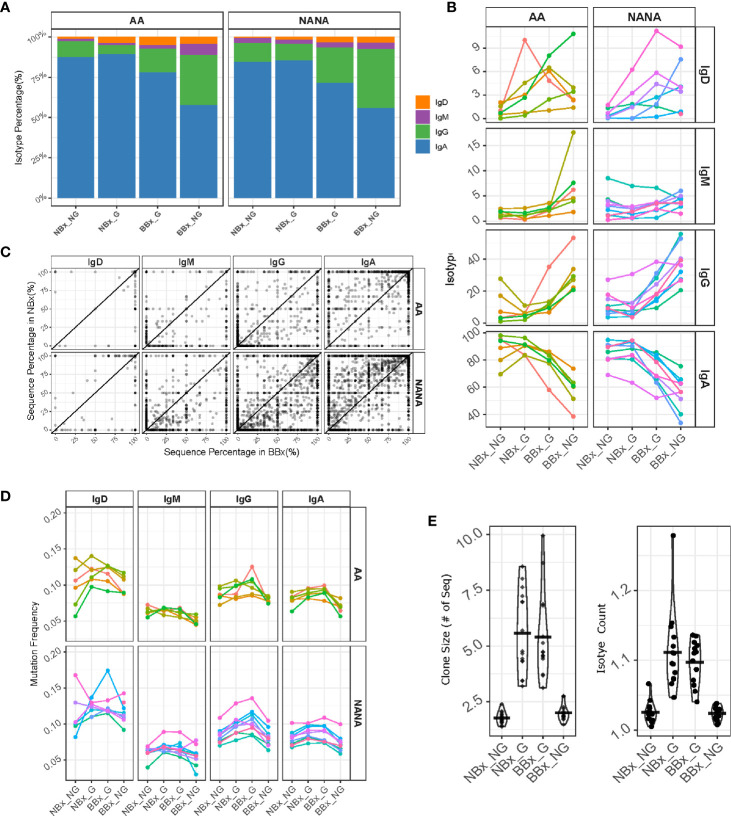
B cells of all isotypes are able to traffic from the nasal to the bronchial mucosa. Global clones are defined as those that contain both nasal and bronchial sequences. We divided the sequences into non-global nasal sequences (NBx_NG) – sequences from clones found in the nasal but not in the bronchial mucosa, global nasal sequences (NBx_G) – the nasal mucosal sequences from global clones, global bronchial sequences (BBx_G) – the bronchial sequences from global clones and non-global bronchial sequences (BBx_NG) – sequences from clones found only in the bronchial mucosa. **(A)** The percentages of the total number of sequences belonging to each isotype (IgD = orange, IgM = purple, IgG = green and IgA = blue) were plotted for each of the four sequence groups for atopic asthmatics (AA) or non-atopic, non-asthmatic controls (NANA). The percentages were calculated for each subject first and then averaged for the asthmatics and non-atopic controls separately. **(B)** Linked changes in the percentage of each isotype from NBx_NG to NBx_G to BBx-G and BBx_NG were plotted for each individual (each line represents an individual, colored the same way as panel **(D)**. **(C)** For each global clone, corresponding to a data point in the plot, the percentages of sequences of a given isotype within the clone found in the nasal mucosa (y-axis) were plotted against the percentages of sequences from the bronchial mucosa (x-axis). **(D)** The mutation frequencies were plotted as the mean of the mutation frequency for sequences of a given isotype for each individual (each line represents an individual, colored the same way as panel **(B)**. **(E)** Left: mean clone size for a given cell subset in each subject for non-global and global clones from either the nasal or bronchial mucosa. Right: mean numbers of isotypes in a clone (right). Each point represents an individual.

To analyze the effect of trafficking within the respiratory tissue, we divided the clones and sequences into non-global nasal sequences (NBx_NG) – sequences from clones found in the nasal but not in the bronchial mucosa, global nasal sequences (NBx_G) – the nasal mucosa sequences from clones that spanned the nasal and bronchial mucosa, global bronchial sequences (BBx_G) – the bronchial sequences from clones that spanned the nasal and bronchial mucosa and non-global bronchial sequences (BBx_NG) – sequences from clones found in the bronchial but not nasal mucosa. We used the previous evidence of predominant directional trafficking from the nasal to the bronchial mucosa to track selection working on B cell clones leaving the nasal mucosa and clones entering the bronchial mucosa. We first observed that the B cell clones leaving the nose (NBx_G) are similar to the ones only found in the nasal mucosa (NBx_NG) in terms of isotype composition with a mean of 86% IgA, 10% IgG and smaller amounts IgD and IgM (**Figures 6A, B**). The values were similar in atopic asthmatics and non-atopic controls. However, we found a shift in isotype composition towards more IgG and less IgA in the B cells clones entering the bronchial mucosa (BBx_G) (mean of 74% IgA and 19% IgG), which was even more distinct in the clones found only with the bronchial mucosa (BBx_NG) (mean of 57% IgA but 34% IgG) (**Figures 6A, B**). This trend for a shift in isotype from IgA to IgG was replicated within single B cell clones when comparing the fractions in the nasal and bronchial mucosa, consistent with a possible scenario of B cell migration in concert with local proliferation and class switch recombination (CSR) from the nasal to the bronchial mucosa (**Figure 6C**). Our sequence analysis does not allow distinction of individual immunoglobulin sub-classes, but given the relative 5’ to 3’ position of the IGHC gene-segments in the immunoglobulin gene-locus and the direction of switching, this would be limited to switching from IgA1 to IgG2 or IgG4. This picture was most distinct in the non-atopic controls.

Somatic hypermutation takes place in activated B cells. The overall mutation frequency was significantly higher in the global clones (both the nasal and bronchial mucosa fractions; NBx_G and BBx_G) compared to the non-global clones from both tissues (NBx_NG and BBx-NG) for IgA and IgG (p<0.05; **Figure 6D**) with the exception of IgG clones from the nasal mucosa of asthmatics. For IgD and IgM clones the picture was mixed with significantly more mutations in global IgD and IgM clones in the bronchial mucosa of asthmatics (BBx_G) and significantly more mutation for nasal global (NBx_G) IgM clones in non-atopic controls (p<0.05; **Figure 6D**). This strongly suggests the possibility of site-specific, isotype-specific variation in B cell mobilization. Compared to local sequences from both the nasal and bronchial mucosa, global B cell clones were significantly larger and tended to comprise greater numbers of isotypes (**Figure 6E**), consistent with the hypothesis that global clones arise from activated B cells.

**Figure 7:**
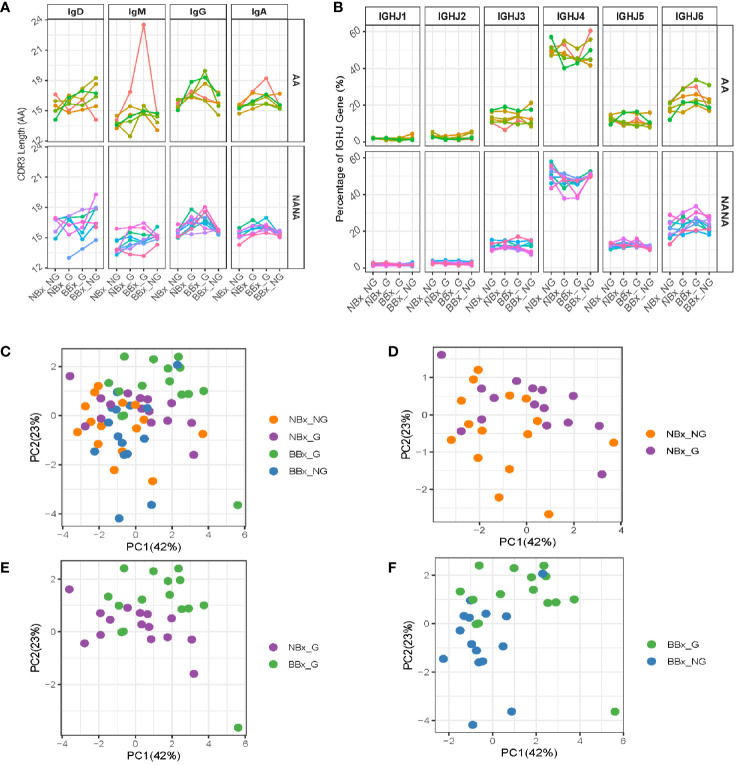
Global clones have longer CDR3 regions with distinct amino acid properties. Sequences from the nasal and bronchial mucosa were divided into groups as described in **Figure 6**. **(A)** The mean lengths (in amino acids) of the CDR3 region of sequences belonging to each group were calculated for each individual (each line represents an individual, colored the same way as panel **(B)** and **Figure 6**). **(B)** The IGHJ-gene usage in sequences of the four sample types was plotted for atopic asthmatics (AA) and non-atopic, non-asthmatic controls (NANA) separately for each individual (each line represents an individual, colored the same way as panel **(A)** and **Figure 6**). Eight different properties of the CDR3 amino acids were calculated (see **Supplementary Figure 7**) and summarized in PCA plots. Each unique sequence was included with the weight of one. The PCA plots were for **(C)** the four sequence subsets together (non-global nasal sequences (orange), global nasal sequences (purple), global bronchial sequences (green) and non-global bronchial sequences (blue), **(D)** non-global (orange) versus global (purple) nasal sequences, **(E)** global nasal (purple) versus bronchial (green) sequences and **(F)** global (green) versus non-global (blue) bronchial sequences.

To look for evidence of differences in selection between the tissues, we further analyzed the heavy chain 3^rd^ complementarity determining region (CDR3). This region is not only the most variable region of an antibody including significant variability in length, but it is also usually critical for antigen binding and therefore a major site for affinity maturation. We found that the CDR3 length was significantly increased in both nasal and bronchial global sequences of IgA and IgG isotype (p<0.05 for all comparisons except IgA from the bronchial mucosa in asthmatics) (**Figure 7A**). This may, at least in part, reflect increased usage of the longest IGHJ gene, IGHJ6, and reduced usage of the shorter IGHJ4 gene in global sequences, as compared with both nasal and bronchial non-global clones, although this didn’t attain statistical significance (**Figure 7B**). The IgM CDR3 length was consistent between both subsets of non-global and both subsets of global B cell clones (with the exception of one asthmatic outlier). We also note that the CDR3 length increased in IgD, so that the non-global bronchial mucosal IgD clones displayed the longest CDR3 (**Figure 7A**, p=0.034 for NANA), suggesting differences in selection of IgD clones with nasal and bronchial mucosal origin. These observations suggested that some selective forces were influencing which B cells could leave the nasal and enter and/or ultimately survive in the bronchial mucosa. To determine whether the differences in CDR3 length were associated with other physicochemical properties, we analyzed eight amino acid properties of the CDR3 (see **Supplementary Figures 7A–H**). PCA analysis based on the CDR3 amino acid properties showed a separation between the four clonal subsets (non-global and global nasal sequences and non-global and global bronchial sequences) (**Figures 7C–F**). The separations are particularly distinct between global clones in the nasal and bronchial mucosa (**Figure 7E**) and between global and non-global clones within the bronchial mucosa (**Figure 7F**) suggesting that some selective pressure is found in the trafficking between the nasal and the bronchial mucosa and *in to* the bronchial mucosa. This selection also operates on the isotype (**Figure 6B**), mutational load (**Figure 6D**), the CDR3 length (**Figure 7A**) and IGHJ-gene usage (**Figure 7B**) showing that the repertoire is progressively shaped as B cells traffic from the upper to the lower respiratory tract.

### A Diverse Subset of Heavily Mutated IgD-Only B Cells With Long CDR3 Regions Are More Prevalent in the Bronchial Mucosa of Asthmatic Patients Compared With Non-Atopic Controls

**Figure 8:**
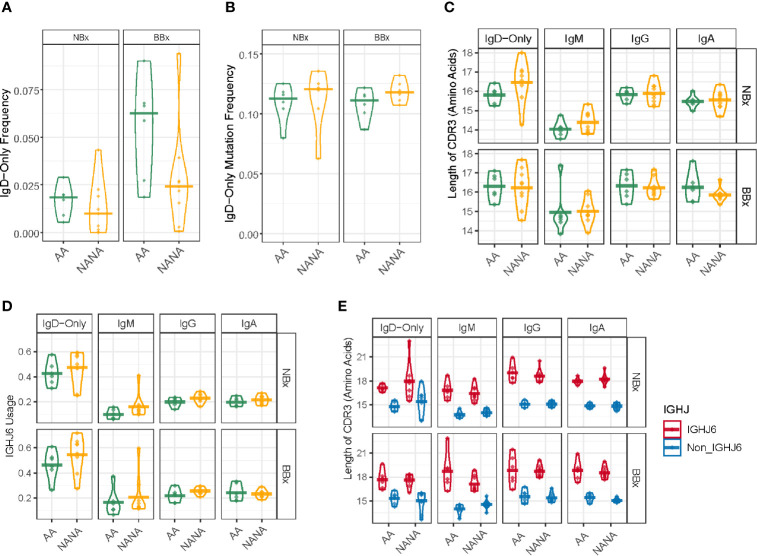
Highly mutated IgD-only sequences with a long CDR3 region and a bias towards IGHJ6 are found in both the bronchial and nasal tissue. **(A)** IgD-only sequences are more commonly found in the bronchial mucosa (BBx) of atopic asthmatics (AA) compared to non-atopic controls (NANA). Lower frequencies of IgD-only sequences are seen in the nasal mucosa (NBx) of both subject groups. NANA03, 07 and 12 have been excluded from this and all subsequent analyses involving the IgD-only cell subset as they have too few IgD-only sequences (< 20 IgD-only sequences). **(B)** IgD-only sequences have a high rate of somatic hypermutation in both atopic asthmatics and non-atopic controls and both bronchial and nasal mucosa. No significant differences were found between groups. **(C)** The CDR3 lengths (given as the numbers of amino acids) of sequences belonging to each sequence type (IgD-only, IgM, IgG and IgA). Each dot represents the sequences from one individual. The IgD-only CDR3s were significantly longer than those from IgM sequences (p=0.05) except in bronchial biopsies from asthmatics which showed only a trend (p=0.067) but not compared to other isotypes (p>0.05 for all other comparisons). No significant differences were found between atopic asthmatics and non-atopic controls. **(D)** IGHJ6 is over-used in IgD-only sequences in both bronchial and nasal tissue (p<0.05 for all comparisons). Each dot represents an individual and the line is the mean for the group. **(E)** Sequences where divided into those using IGHJ6 or other IGHJ genes (Non_IGHJ6), which shows that the significantly longer CDR3 regions of IgD-only sequences compared to IgM sequences are found even in sequences using other IGHJ genes (blue) (p<0.05 in both the nasal and bronchial mucosa of atopic asthmatics). Each dot represents an individual and the line is the mean for the group.

Whereas the nasal mucosal biopsies yielded a higher overall frequency of B cell clones producing IgA, the bronchial mucosal biopsies yielded a higher frequency of clones producing IgD (**Figure 6A**), 99% of these being IgD-only. IgD-only B cell clones were more prevalent (mean 6.3%) in the bronchial mucosa of the atopic asthmatics compared to the non-atopic controls (mean 2.5%), although this difference did not attain statistical significance (p=0.087) (**Figure 8A**). In contrast, in the nasal mucosa the frequency of IgD-only B cell clones was significantly lower (about 1-2%, p=6.1^e-05^) than in the bronchial mucosa and was similar between the asthmatics and the control subjects (p=0.48). A mean of 20% of the IgD-only clones from each individual asthmatic, and 15% in individuals from the control group, were shared between the nasal and bronchial mucosa. The IgD-only mutation frequency was very high (11-12% on average) in both groups and at both sites (**Figure 8B**), higher than for any other isotype (**Figure 4B**).

We and others (7, 23) have previously reported that the IgD from B cells expressing IgD-only have longer heavy chain CDR3 regions compared to IgD from clones also expressing IgM. In the present study, we found that IgD-only B cell clones in the bronchial and nasal mucosa had significantly longer CDR3s than IgM-sequences (p<0.05) with the exception of bronchial IgD-only sequences from atopic asthmatics where the same trend was present, but the difference was not significant owing to one inexplicable outlier (AA02) (p=0.067). No significant differences were found with IgG (p=0.69) and IgA (p=0.08) (**Figure 8C**). IGHJ6 is the longest of the six IGHJ genes and over-usage is often associated with long CDR3 regions. We found a distinct over-usage of IGHJ6 in the IgD-only sequences compared to the IgM, IgG and IgA isotypes in both nasal and bronchial biopsy samples (**Figure 8D**, p<0.05 for all comparisons). However, this overuse of IGHJ6 could not by itself explain the significantly longer CDR3s of IgD-only compared to IgM, as even IgD-only sequences using non-IGHJ6 genes were significantly longer than their IgM counterparts (**Figure 8E**, p<0.05 for both NBx and BBx from asthmatics).

When analyzing individual amino acid properties, as described comparing non-global and global clones, we found several significant differences (**Supplementary Figure 8**). For example, the CDR3 regions of IgD-only sequences from both bronchial and nasal biopsies had a significantly higher GRAVY index (indicative of a more hydrophobic nature of the side chains) and higher aliphatic index compared to IgM in both asthmatics and non-atopic controls. The IgD-only CDR3 regions also had significantly lower contents of aromatic amino acids compared to the three isotypes tested, IgM, IgG and IgA. In the asthmatic patients this was true both in the nasal and bronchial mucosa whereas it only reached significance in the bronchial mucosa of non-atopic controls. This suggests that IgD-only sequences are selected not only for long CDR3 regions but also for certain properties of the amino acids, which they encode. The more hydrophobic, and in particular aliphatic nature of these side chains, may indicate that the antigenic epitopes that are recognized display a similar nature; such hydrophobic interactions generally make the predominant contribution to protein/protein interactions.

### IgD-Only Cells Show Evidence of Antigenic Selection That Varies Between Tissue and Sample Type

**Figure 9:**
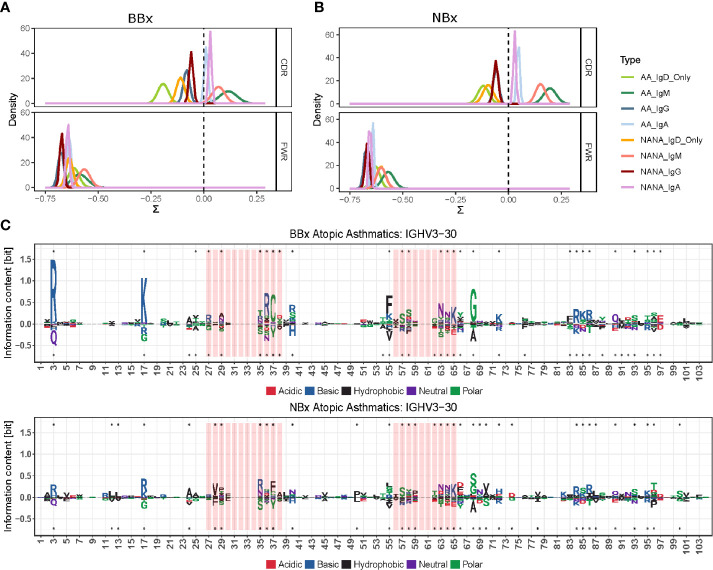
The antigenic selection pressure exerted on IgD-only cell varies between tissues and sample types. **(A, B)** BASELINe estimation of the posterior distribution of selection strengths (∑) for the complementarity determining regions (CDR) and framework regions (FWR) of IgD-only sequences (light green for atopic asthmatics (AA) and yellow for non-atopic, non-asthmatic controls (NANA)] and those of other switched isotypes: IgA (light blue AA, violet NANA) and IgG (dark blue AA, maroon NANA) and IgM sequences (dark green AA, pink NANA) in **(A)** the bronchial mucosal and **(B)** the nasal mucosa samples. IgD-only sequences display significantly more negative selection strength in the CDRs compared to switched isotypes and IgM in both the bronchial and nasal mucosa. The IgD-only bronchial mucosa also showed a statistically significant difference between atopic asthmatics and non-atopic controls with asthmatics displaying the lowest pressure in the CDRs of all sample types tested. **(C)** Position weight matrix (pwm) analyses of somatic hypermutation generated amino acid substitutions selected for in IgD-only sequences (over the axis) and against (below the axis) in the IGHV3-30 gene in the bronchial (top panel) and nasal (bottom panel) mucosa of atopic asthmatics. The red areas indicate CDR1 and 2 and the white areas are the FWRs. The letters denote the one letter amino acid codes and the colors show the properties of the amino acids selected for or against with red = acidic, blue = basic, black = hydrophobic, purple = neutral and green = polar. The size of the letters shows the strength of selective pressure in the given position. Dots indicate significant differences between IgD-only and the other sequences.

To further analyze selection in IgD-only, we performed BASELINe analyses of the selection strength (∑). This revealed that IgD-only B cells from both the bronchial and nasal mucosa showed evidence of a significantly stronger negative selection pressure in the CDR1 and CDR2 regions compared with that of switched isotypes (IgA and IgG) and IgM (p<0.01 for all comparisons) (**Figures 9A, B**). Remarkably, we also uncovered evidence of a significantly elevated selection strength scores in the bronchial, but not the nasal, mucosal IgD-only B cell clones from the non-atopic controls as compared with the asthmatic patients (p=0.01 and 0.28 respectively) (**Figures 9A, B**). No such differences between asthmatics and non-atopic controls were found for the other switched isotypes (p>0.05), but in both tissues the IgM sequences showed significantly higher selection strength in the CDRs compared to the switched isotypes (p<0.00001). All subsets - from both asthmatics and non-atopic control subjects and from both the nasal and bronchial mucosal tissue – showed negative selection strength scores in the framework regions (FWR). This pattern of selection pressure is consistent with antigen-driven selection and it is interesting that these differences in IgD-only selection between the asthmatics and controls were evident only in the bronchial mucosa and not evident for any other antibody isotype.

We previously found that the ratio of replacement to silent mutation rates in the CDRs (a measure of selection strength) was negatively correlated with the number of mutations within a clone (17). This indicated that more mutated clones tended to have a stronger signal of purifying selection; i.e., selection for maintaining the amino acid structure of the BCR. A similar pattern was observed in the present study (**Supplementary Figure 9**). The IgD-only subset tended to have the highest mutation frequency and the most negative selection strength scores compared to all other isotypes. One possible explanation for this relationship is that early replacement mutations are positively selected, but once a significant (optimal) affinity increase is achieved, additional replacement mutations are more likely to be selected against. To explore which amino acid replacements were being selected for (or against) in the IgD-only sequences, we analyzed the amino acid usage at each position in the IGHV gene using position weight matrices (PWMs) (see **Supplementary Materials and Methods**) for the most commonly used IGHV genes (defined as more than 2.5% usage in at least three individuals). Compared to sequences of IgM, IgA and IgG isotype, many positions in the CDRs of IgD-only sequences showed a strong preference for (and against) specific against amino acid changes both in the bronchial and nasal mucosa (example for IGHV3-30 in **Figure 9C** top and bottom, respectively). Interestingly, the FWRs also showed a strong preference for particular amino acids at many positions. Many of these preferences were similar in the two tissues (e.g. positions 86 and 64 in FWR2 and CDR2, respectively, see **Figure 9C**) whereas others were tissue specific (e.g. positions 17 and 37 in FWR1 and CDR2, respectively, see **Figure 9C**). There was, however, a general trend for stronger selection in positions in sequences from the bronchial mucosa compared to sequences from the nasal mucosa (**Figure 9C**) confirming that there are differences in the selective pressures on IgD-only B cells in the lower and upper respiratory tract.

## Discussion

In this study we employed Adaptive Immune Receptor Repertoire (AIRR) analysis to compare the antibody repertoires of B cells at various sites in the upper (nose) and lower (lung) respiratory tract mucosa of atopic asthmatic patients and non-atopic controls in order to gain deeper insight into the mechanisms underlying immune defense and disease pathogenesis in asthma. In summary, our data and analysis clearly demonstrate passage of B cells between the upper and lower respiratory tract mucosa, dominantly from the nasal to the bronchial mucosa, and mutually between the mucosal surfaces of the right and left bronchial trees. Our observations that B cell clones undergo somatic hypermutation and may undergo isotype switching during the trafficking suggest that it is predominantly activated B cells that traffic. We have also uncovered evidence of site- and maturation-specific selective forces shaping the local B cell repertoire. Furthermore, we confirm the presence of a special subset of IgD-only B cells, most prominent in the asthmatic bronchial mucosa, characterized by a very high degree of somatic hypermutation and with features of selection pressures distinct from those operating on the B cells of other isotypes.

We have further clarified the practicability of determining the B cell repertoire in the respiratory mucosa from a limited number of mucosal biopsies from the upper and lower respiratory tracts. This corresponds to our previous findings (7, 24), but here we also show that sampling only one lung enables a fair, if incomplete, representation of the total B cell repertoire of the bronchial mucosa tissue (**Supplementary Figure 1**). We also analyzed the B cell repertoire in the peripheral blood. This was less complete than that of the bronchial mucosa, but sufficient to demonstrate that the peripheral blood is a possible route by which B cells are disseminated to other sites in the respiratory tract.

We found that B cell clones could be shared between all of the sites that we sampled, *i.e.* the nasal mucosa, four different sites within the bronchial mucosa ranging from the carina down to the 4^th^ generation bronchi of the lower lobe as well as the peripheral blood. There was no detected directional bias for trafficking between the two lungs but clones shared between the left and right bronchial mucosa were more likely to be related to cells in the nasal mucosa, suggesting that the shared clones were selected for mobilization, most likely following activation by exposure to specific antigen (25). The biological consequence of mobilization of B cell clones would be uniform sensitization to antigens across the respiratory tract following exposure at any site. This would likely facilitate immune defense as well as pathological processes, which was also previously suggested by the “united airways disease” model (6). This could be an advantage in terms of immunological protection but clearly disadvantageous in atopic disease, in that antigen sensitization in one tissue could lead to reaction in another.

Despite many shared clones, the B cell repertoire was more diverse in the nasal compared with the bronchial mucosa. Diversity was also higher in the bronchial (but not nasal) mucosa of non-atopic control subjects compared with the atopic asthmatics (**Supplementary Figure 3**). It is tempting to interpret this observation in relation to the function of B cells in adaptive immunity and/or asthma pathology. As a result of filtering and limitations of particle size in the upper airways, the bronchial mucosa is likely to be exposed to fewer, but conceivably more relevant antigens against which local antibodies, with at least adequate specificity, affinity and isotype, are directed. The more focused repertoire in the patients with atopic asthma compared with the non-atopic controls may reflect differences in the local microbiome and possibly inappropriate (pathological) activation of only limited B cell subsets like IgD-only B cells found to be up-regulated in asthmatics (see below). Another example, although they could not be detected in this study owing to technical issues, would be B cells expressing IgE, also known to be up-regulated in the bronchial mucosa of asthmatics (11).

With regard to isotype distribution, we found IgA to be the dominant antibody isotype in the nasal mucosa. This may reflect an evolutionary adaptation to the local environment, considering that IgA antibodies are secreted into the airways lumen and inhibit contact of their targets with the mucosa. In contrast, although IgA was still the most frequent isotype in the bronchial mucosa, this bias was diminished and compensated by the higher proportions of IgM, IgG and IgD clones lower down in the respiratory tract, where direct contact of the mucosa with particulate antigens is progressively less likely. Since IgA clones cannot undergo isotype switching to IgM, IgD, IgG1 or IgG3, this suggests activation of new cells/clones in the bronchial mucosa. Our sequences do not allow individual sub-class identification, so the observed change from IgA to IgG that was even found in individual clones spanning the nasal and bronchial mucosa (**Figure 6C**) must be from IgA1 to IgG2 or IgG4 as these are the only possible switches due to the location of the individual gene-segments on the chromosome. These findings implicate selective trafficking of certain clones between the two tissues. It was not possible to determine whether CSR occurred *en route* to the bronchial mucosa or following entry, but selection against IgA did not seem to occur at the stage of clones leaving the nasal mucosa. Our observation that global clones tended to have a greater mutational load (driven by SHM and affinity maturation) is fully compatible with a scenario of antigen-driven migration of B cells which have undergone proliferation, isotype switching by CSR and affinity maturation. However, as for CSR, it was not possible to determine whether SHM took place *en route* to the bronchial mucosa, for example in local, draining lymph nodes or other systemic lymphoid tissue, or locally within the mucosa itself. We also searched for directional bias in B cell migration using a parsimony-based phylogenetic test, which takes into account the progressive accumulation of mutations from the germline toward the tips of the clonal linage trees (16). This showed that trafficking of B cell clones between the two lungs is bi-directional and not significantly biased in either direction, whereas there was significant biased trafficking from the nasal to bronchial mucosa. The signature of raised levels of SHM was significantly stronger in the atopic asthmatic patients compared with the non-atopic controls, which might suggest an overall higher degree of B cell activation and directional trafficking in the asthmatic subjects. Of interest, 5 of the 6 asthmatics but none of the 9 control subjects reported suffering from rhinitis consistent with the United Airway Disease Hypothesis (6). We sampled the nasal mucosa biopsies 4-43 days before the bronchial mucosa biopsies and we found that the time between the sampling did not affect the conclusions about directional B cell trafficking.

Our data, while clearly confirming B cell migration throughout the respiratory tract mucosa, do not make it possible precisely to determine the routes of this migration. However, our finding of some clones with members in both tissues and the peripheral blood suggests that the peripheral blood may be involved in the trafficking. Trafficking may however also have included the lymphatics followed by homing back to local lymph nodes and thence back into the lymphatics, or homing to, and migration through local, mucosal blood capillaries or both. This implies a degree of B cell “homing”, which is intriguing because, so far as we are aware, there is no established mechanistic basis for this. Sampling of local lymphoid aggregates in the context of mucosal inflammatory diseases (“iBALT”) (2) might conceivably shed further light on this conundrum in future studies. There is some precedent for this, as we have previously established that in multiple sclerosis accumulation of SHM takes place in the draining cervical lymph nodes from where the B cells can traffic to the central nervous system (26). A complicating factor for local B cell maturation within the bronchial tissues itself is that, although nasal and bronchial mucosal B cells have been documented in previous studies to express markers of recent SHM (27), the duration and time course of expression of these markers in relation to the dissemination of clones in the respiratory tract remain to be clarified.

The IgD antibodies expressed by the B cells in the respiratory tract mucosa differ from those in PBMCs, which are expressed principally by mature, naive IgM^+^IgD^+^ B cells that produce both IgM and IgD with identical VDJ rearrangements through alternative mRNA splicing. These B cells have not yet “experienced” antigen activation, SHM and affinity maturation or CSR, and the vast majority will never do so or survive long enough to be observed. In contrast, the antibodies from IgD-only B cells that we have identified in the bronchial, and to a significantly lesser extent the nasal mucosa, in this and our previous study (7) differ from those of other isotypes. They are more highly mutated (7, 10), and have CDR3s that are significantly longer than those of IgM cells - but similar in length to other switched isotypes – and show greater than two-fold increase in usage of the IGHJ6 gene segment. IgD-only B cells arise by non-canonical switch recombination (9) and are found in both the asthmatic patients and the non-atopic control subjects but are up-regulated in the asthmatics. Compared to other isotypes, bronchial mucosal IgD-only B cells show significantly stronger signature of purifying (negative) selection in the CDR1 and 2 regions (**Figures 9A, B**), particularly in asthmatics. It has previously been reported (17) that clones with large numbers of mutations show a stronger signature of purifying selection. This was hypothesized to be because the benefit of new replacement mutations is expected to decrease once a sufficiently high level of affinity has been reached. This seems possible here as well, because IgD-only clones generally have a high level of SHM. On the other hand, continual exposure and affinity maturation against the same or limited antigens (perhaps from commensal bacteria) could also explain why IgD-only B cells have strong signatures of purifying selection, although a different reason for lower selection pressure in the IgD-only cells cannot be ruled out (see below). The really interesting picture that emerges from our analysis is that CDR selection pressure in asthmatics and non-atopic controls of IgD-only clones differs in the bronchial, but not in the nasal mucosa, suggesting that the selective forces (antigens) driving the affinity maturation are different in the two tissues. This is consistent with the identified differences in the local microbiomes in the upper and lower airways (28) and with the concept of filtering and limitations of particle size when moving down the airways referred to above.

Conservation of sequence is, of course, far greater in the framework regions of all antibody isotypes, since these regions maintain the structural framework in which the CDRs are presented. Surprisingly, however, using position weight matrix analyses we found that some of the IgD-only clones utilizing certain IGHV genes exhibited a particular pattern of framework mutations. Many of these mutations differed from those of antibodies of IgM, IgA or IgG isotypes, and we found differences between the nasal and bronchial mucosa, again suggesting that differences in local stimuli may be reflected in differences in mutation patterns. Such patterns of framework mutations as seen in our IgD-only cell subsets are found in antibodies that to bind to superantigens (29–31).

Having analyzed the FWR and CDR1 and 2, we wanted to focus on the CDR3 region known to be the most important region for antigen-binding. We found that, similarly to other switched isotypes, IgA and IgG, IgD-only antibodies have significantly longer CDR3 regions compared to IgM antibodies. IgD-only antibodies also show a distinct bias towards the expression of the longest of the IGHJ-genes, IGHJ6, which could explain the long CDR3s, however we found that the 5’ ends of the IGHJ6-gene were heavily trimmed in the IgD-only rearrangements (data not shown). IGHJ6 harbors 5-6 tyrosine residues at the 5’ end and since tyrosine residues are the amino acids most commonly involved in antibody-antigen interactions because of their ability to recognize and interact with antigens both by hydrogen bonding and hydrophobic interactions (32, 33), untrimmed IGHJ6-gene segments could be hypothesized to contribute to CDR3s with strong antigen-binding potential. However, consistent with the heavy trimming, we found a clear and significant selection against aromatic amino acids (**Supplementary Figure 8**) in the CDR3 regions of IgD-antibodies using IGHJ6. We also found higher hydrophobicity (GRAVY index) and higher aliphatic contents of IgD-only CDR3 (**Supplementary Figure 8**). Taken together, this suggested that IgD-only CDR3s were dominated by more aliphatic amino acids (alanine, valine, leucine and isoleucine) and fewer aromatic amino acids (tyrosine, phenylalanine and tryptophan), which is a pattern of amino acid composition that is inconsistent with antigen selection and specificity. In support of this, the BASELINe analyses showing that the selection strength in the CDR1 and 2 of IgD-only sequences was significantly more negative than that of other isotypes, especially in the bronchial mucosa, can also be interpreted as lack of evidence for antigenic selection. This is an alternative, but not mutually exclusive, interpretation of the negative selection pressure being caused by a very high level of SHM, as previously discussed.

In light of our results and reports from clinical studies (34, 35), we suggest that commensal bacteria in the lower and/or upper respiratory tract or recurrent bacterial infections associated with asthma could be involved in driving mutations in the IgD-only cells either in a B-cell superantigen-like manner (explaining the underrepresentation of aromatic side chains in the CDR3) or towards purifying selection due to antigens of aliphatic nature to match the over-representation of aliphatic and hydrophobic side chains in the CDR3. The commensals could also be driving the generation and proliferation of IgD-only cells. There is some precedent for this, as switching to IgD-only in a mouse model has been shown to be stimulated by the microbiota and to occur only in mucosa-associated lymphoid tissue (8). The effect could be restricted to certain IGHV-gene (families) as previously reported for B cell IGHV-family gene expression in antibodies driven by certain enterotoxins from the commensal *Staphylococcus aureus* (36, 37), even though these enterotoxins are better known as T cell superantigens. Staphylococcal protein A (SPA) is also known to specifically bind to a specific IGHV-gene family (IGVH3) (31). Two of the study subjects, one asthmatic and one control, had clinically relevant serum concentrations of IgE antibodies specific for the *S. aureus* enterotoxin toxic shock syndrome toxin-1, TSST-1, as detected by ImmunoCAP, and three other individuals (two asthmatics and one control) had detectable serum concentrations of anti-enterotoxin-specific IgEs, evidence that *S. aureus* had been present in those subjects.

Two species of common commensal bacteria, *Moraxella catarrhalis* and *Haemophilus influenzae* express IgD-binding proteins that can bind to the Cδ1 domain of IgD on naïve IgD-expressing B cells (38, 39), thereby acting as superantigens by stimulating polyclonal B cell proliferation and immunoglobulin class switching (40, 41). The very same bacteria are associated with asthma (34, 35). It is therefore plausible that these commensal bacteria may be involved in driving the generation of IgD-only B cells but, given the findings of this study examining the variable domain of IgD-only B cells, we propose that other bacteria/superantigens might also be involved. While analysis of these hypotheses is beyond the scope of the present study, the data lead us further to hypothesize that IgD has evolved to provide a combination of innate interaction with bacteria through the Cδ-1 region as well as adaptive immunity to bacteria through SHM and selection in the variable regions as we show here. The cost of this is likely increased inflammation and the possibility of autoimmunity by the selection of autoantibodies (23). While these observations are currently speculative, we trust that they may uncover new possible mechanisms of bronchial inflammation in asthma.

## Data Availability Statement

The datasets presented in this study can be found in online repositories. The names of the repository/repositories and accession number(s) can be found below: https://www.ncbi.nlm.nih.gov, PRJNA698997.

## Ethics Statement

The studies involving human participants were reviewed and approved by London Central Ethics Committee, REC number 15/LO/1800. The patients/participants provided their written informed consent to participate in this study.

## Author Contributions

LO-L and HJG designed the research. CJC, RL and EBC collected the samples. LO-L, BSS and NEGU processed the samples. YJ, CC, TGJ and RH performed the sequencing and initial data processing. HM, KBH, NN and SHK analyzed the data and produced the figures in discussion with LO-L and HJG. LO-L, HJG, SHK and CJC. wrote the paper. All authors contributed to the article and approved the submitted version.

## Funding

LO-L, BS, NU, CC and HG were supported by a research grant from The Medical Research Council, UK (grant number MR/M022943/1) and by the National Institute for Health Research (NIHR) Clinical Research Facility at Guy’s & St Thomas’ NHS Foundation Trust and NIHR Biomedical Research Centre based at Guy’s and St Thomas’ NHS Foundation Trust and King’s College London. The work of SK was supported in part by the National Institute of Allergy and Infectious Diseases of the National Institutes of Health (award number R01AI104739). The work of HG and LO-L was also supported by the award of a Leverhulme Emeritus Professorship to HG (award number EM-2019 027).

## Author Disclaimer

The views expressed are those of the authors and not necessarily those of the NHS, the NIHR or the Department of Health.

## Conflict of Interest

SHK receives consulting fees from Northrop Grumman. KBH receives consulting fees from Prellis Biologics. YJ, CC, TGJ and RH are employed by Bristol Myers Squibb.

The remaining authors declare that the research was conducted in the absence of any commercial or financial relationships that could be construed as a potential conflict of interest.

The handling editor has declared past collaborations with one of the authors SHK at the time of this review.

## Publisher’s Note

All claims expressed in this article are solely those of the authors and do not necessarily represent those of their affiliated organizations, or those of the publisher, the editors and the reviewers. Any product that may be evaluated in this article, or claim that may be made by its manufacturer, is not guaranteed or endorsed by the publisher.
