# Supplementary material for: B Cell Mobilization, Dissemination, Fine Tuning of Local Antigen Specificity and Isotype Selection in Asthma
Source: Front Immunol. 2021 Oct 14;12:702074. doi: 10.3389/fimmu.2021.702074 (PMC8552043; doi:10.3389/fimmu.2021.702074)
Supplement: Supplementary file 1 [file DataSheet_1.docx]

Supplementary Material

# Supplementary materials and method

## Sequencing data processing

***Bulk (AbSeq) BCR sequencing data.*** pRESTO v0.5.7 (Vander Heiden et al., 2014) was used to perform all stages of raw sequence processing prior to alignment against reference germline sequences. Poor-quality reads (Phred score < 20) and reads aligned with PhiX174 reference genome were removed. Reads were then aligned against template switch sequences and constant region primers, with a maximum mismatch rate of 0.2. A single consensus sequence was generated for each UMI barcode and consensus sequences with only one single contributing read were discarded. Each paired-end UMI consensus sequence was assembled into a full length Ig sequence.

***Single Cell (AbPair) BCR sequencing data.*** The raw fastq files were processed using pRESTO 0.5.3 (Vander Heiden et al., 2014). For each droplet & molecular barcode combination, the number of reads was down-sampled to the 50 highest quality reads prior to consensus calling and assembly. The heavy chain sequences from single cell data were extracted and used for further data analysis.

The heavy chain sequences from bulk and single cell data were combined for subsequent analysis using methods from the Immcantation framework (http://immcantation.org). Initial assignment of V(D)J germline gene annotations were performed using IgBLAST v1.9.0 (Ye et al., 2013) against the International Immunogenetics Information System (IMGT) database (Alamyar et al., 2012), and subsequently processed using Change-O v0.4.0 (Gupta et al., 2015). Non-functional sequences, *i.e.* sequencing in which the IGHJ gene was out of frame, were also removed from the data, as were potential chimeras identified as sequences with more than six mismatches from the germline IGHV gene in a 10-nucleotide stretch. Sequences were first separated into groups with the same IGHV gene, IGHJ gene and junction length. The junction region was defined as the IMGT CDR3 plus the two flanking amino acids (the conserved cysteine on the 5’ side, and the phenylalanine or tryptophan on the 3’ side). The Hamming distance between each sequence to its nearest non-identical sequence was then determined. A histogram of these distances was manually inspected to determine a Hamming distance (normalized by junction length) of 0.18 as a threshold for assigning clonal groups following single-linkage hierarchical clustering. Sequence coverage curve was drawn for each individual sample using iNEXT (Hsieh et al., 2016) to check whether the current sequence coverage had fully saturated the diversity (Supplementary Figure 1).

## Clonal diversity analysis

Clonal diversity was computed using the rarefyDiversity function from Alakazam v0.2.10 with uniform resampling to correct for sequencing depth. The Shannon diversity was calculated at q (the diversity order) =1 and the Simpson diversity indices were calculated at q=2.

## Isotype assignment

Based on the set of known isotype and sub-isotype alleles as listed in the IMGT database, we derived a list of isotype signature sequences from the IMGT IGHC region consensus reference sequences (Alamyar et al., 2012) to assign isotypes to each sequence. These signature sequences were 16 bp long and found at the 5’ end of the constant region upstream of the constant region primers. Using “MaskPrimers” in the pRESTO package (Vander Heiden et al., 2014), the signature sequences were aligned against the IGHC region of each sequence and the isotype was assigned with a maximum mismatch rate of 0.3.

## Calculation of mutation frequency

For each sequence, the mutation frequency was calculated as the number of mutations over the total number of positions of IGHV segment up to nucleotide position 312 (codon 104). The median value for all sequences belonging to a given clone was used to calculate the overall mutation frequency for that clone. We have adopted this approach to avoid skewing of the result by larger clones.

## Generation of clonal lineage trees

Sequences with the same isotype, junction length and IGHV and IGHJ sequences from within the same clone of the same sample were combined and annotated by the function makeChangeoClone of Alakazam v0.2.10 (Gupta et al., 2015). Lineage trees were inferred via maximum parsimony with PHYLIP version 3.697 (Felsenstein, 1989). The analysis of lineage tree topologies was performed using standard graph traversal algorithm provided by the igraph R package version 1.9.0 (Csardi, 2006).

## Selection analysis

The selection strength (∑) on clonal lineages was quantified with BASELINe (Uduman et al., 2011) implemented in SHazaM v0.1.9 (Gupta et al., 2015), using the "HH_S5F" model (Yaari et al., 2013) as the underlying somatic hypermutation targeting model from which expected mutation rates are derived and the local test statistic (Yaari et al., 2012). Each isotype from a clonal lineage was represented by the most highly mutated sequence of that isotype present in the clone. The probability density functions (PDF) for the selection strength on the complementarity determining region (CDR) and the framework region (FWR) were computed by unweighted convolution of the PDFs for individual clones corresponding to the respective regions.

## Amino acid physicochemical property analysis

Multiple amino acid physicochemical properties of CDR3 regions were obtained with the function aminoAcidProperties in the package of Alakazam v0.2.10 (Gupta et al., 2015). The available properties included length (total amino acid count), gravy index (grand average of hydrophobicity), bulkiness (average bulkiness), polarity (average polarity), aliphatic index (normalized aliphatic index), charge (normalized net charge), acidic content (acidic side chain residue content), basic content (basic side chain residue content) and aromatic content (aromatic side chain content).

## Phylogenetic analysis of B cell trafficking patterns

A previously described parsimony-based phylogenetic test was used to estimate B cell trafficking patterns between tissues (Hoehn et al., 2020). Briefly, phylogenetic tree topologies and branch lengths were estimated using maximum parsimony using PHYLIP v 3.967 (Felsenstein, 1989). Given the tissue of origin for each sequence within the tree, a maximum parsimony algorithm was used to reconstruct the most parsimonious set of internal node tissues that required the fewest number of migrations along the tree. Given the set of internal node locations, the proportion of each type of tissue location switch along the tree (switch proportion, SP) was calculated. This process was then repeated on the same tree with randomized tissue locations. The difference between SP calculated on the observed and randomized tree was denoted 𝛿. Mean 𝛿 > 0 indicated a greater proportion of changes in the specified direction in observed trees, while mean 𝛿 < 0 indicated the opposite. To calculate the significance of 𝛿 while accounting for uncertainty in tree topology, this process was repeated for 100 phylogenetic bootstrap replicates (Felsenstein, 1985). Briefly, for each clone i) nucleotide sites were sampled with replacement, ii) tree topologies and branch lengths were estimated as above iii) observed and randomized SP were calculated, and iv) this process was repeated for 100 bootstrap/permutation replicates. The p-value was calculated as the proportion of replicates in which 𝛿 ≥ 0. For a repertoire of trees, this process was repeated, however SP was calculated as the proportion of each switch type across all trees within the repertoire.

## Position Weight Matrix Analysis

A position weight matrix (PWM)-based approach was used in order to calculate amino acids’ rate of growth between non-IgD-only and IgD-only clones at each position within IGHV sequences.

Aligned sequences from non-IgD-only and IgD-only clones were each used to construct a position probability matrix (PPM) by: (1) creating a position frequency matrix (PFM) by counting the occurrences of each amino acid at each position, and (2) creating the PPM by dividing that amino acid count at each position by the number of sequences, thereby normalizing the values. Fold changes were calculated as the log2 ratio of each amino acid frequency from IgD-only-PPM divided by corresponding amino acid frequency from non-IgD-only-PPM. Finally, each fold change was multiplied by the frequency of amino acid from IgD-only-PPM to calculate the conditional information content of the given amino acid in IgD-only clones relative to non-IgD-only clones. Each PWM had one row for each symbol of the amino acid and one column for each position in the IGHV sequence. To make robust calculations, the PPM matrices were only built in cases where there were at least 50 unique IGHV sequences for each of them. Furthermore, to avoid biases created by somatic hypermutation hotspot targeting, the non-IgD-only matrix was constructed from sequences that were randomly sampled so that their mutation distribution matched that of the IgD-only clones.

# Supplementary Figures and Tables

## Supplementary Figures

**
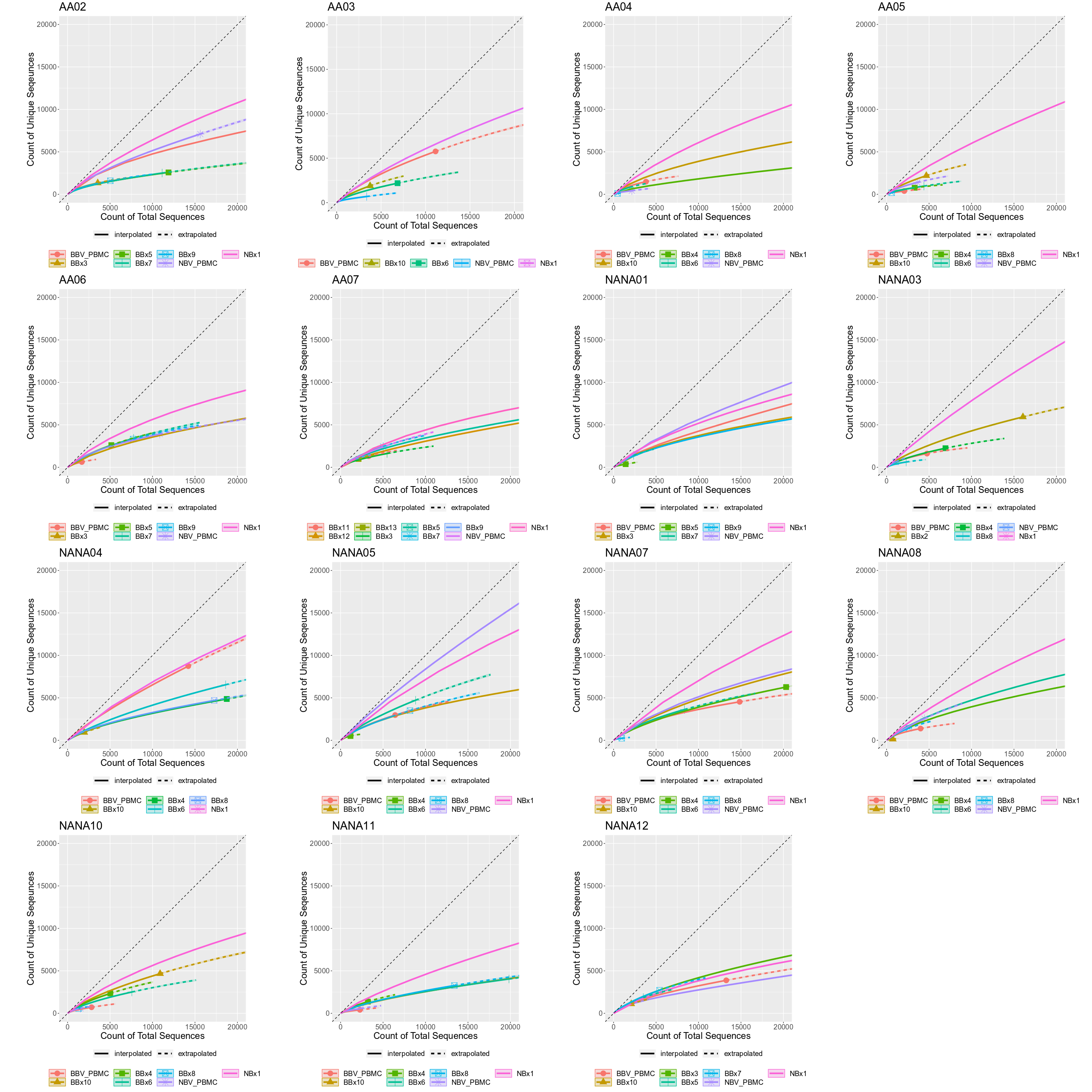
3**

**Supplementary Fig. 1** iNEXT plots (iNterpolation and EXTrapolation) (Hsieh et al., 2016) confirm acceptable coverage and extrapolated total sample sizes for bronchial biopsies (BBx; green, blue, turquoise and olive), nasal biopsy (NBx1; pink) and PBMCs (NBV_PBMC; purple and BBV_PBMC; red) from each of the 15 subjects. Although none of the coverage curves had flattened completely, indicating diversity saturation (which would not be expected given its extent, particularly in PBMC (Soto et al., 2019)), they all showed evidence of flattening, indicating coverage to a degree where sequences had been repeatedly captured


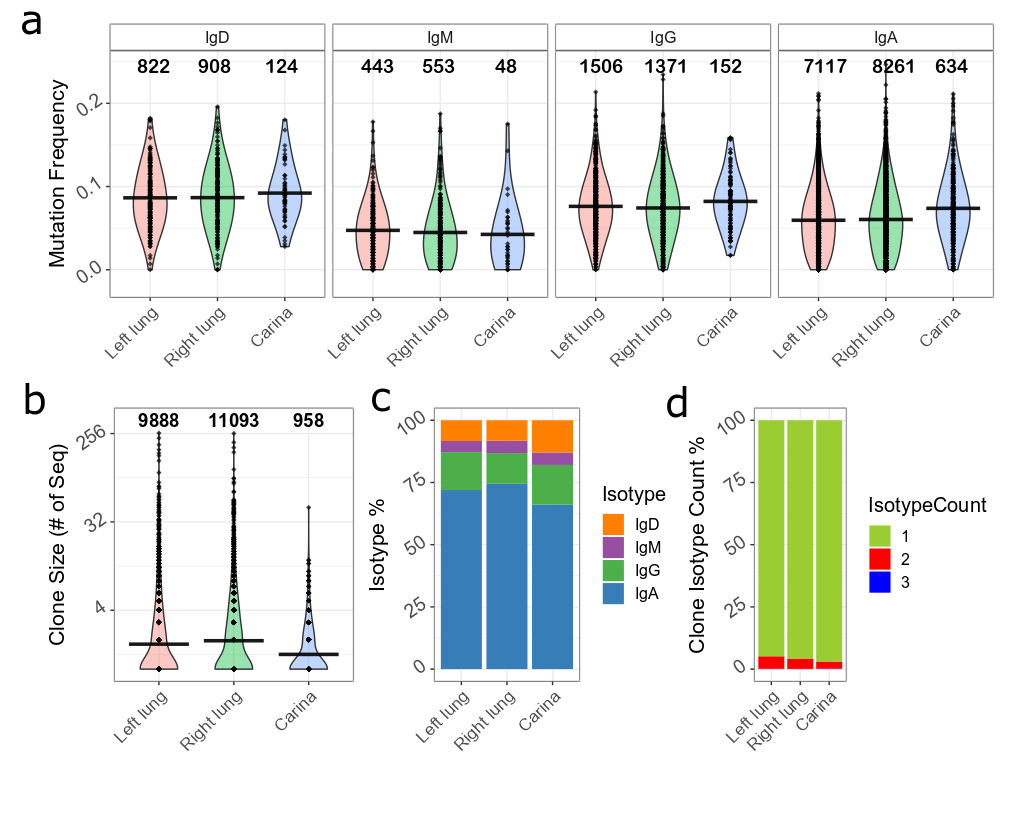


**Supplementary Fig. 2** Sequences from biopsies of the left and right bronchi as well as the carina from AA07 show similar mutation frequencies, clone size and isotype distribution**. a)** Mean isotype-specific mutation frequencies from each clone from the left lung bronchial biopsies (Left lung), right lung biopsies (Right lung) or the biopsies from the carina plotted as a violin plot. Each dot represents a clone and the number on top of each violin indicates the number of clones from each site. **b)** Numbers of sequences per clone from the given site. The numbers on top of each violin indicate the number of clones at each site and the horizontal lines indicate the means. Bar plots showing **c)** percentages of the total sequences of a given isotype and **d)** the percentage of the clones containing a given number of isotypes. Sequences from the left and right bronchial mucosa are indistinguishable in all these terms. The data pertaining to the sequences from the carina (slightly higher mutational load (except for IgM), more IgD clones but slightly fewer total clones) are not significantly different and likely attributable to the fact they originated from a single sample, compared with three each from the right and left bronchial mucosa, and that 68% of the carina sequences are from expanded clones (clones spanning more than one site) compared to 47% and 44% from the left and right lung mucosa, respectively.


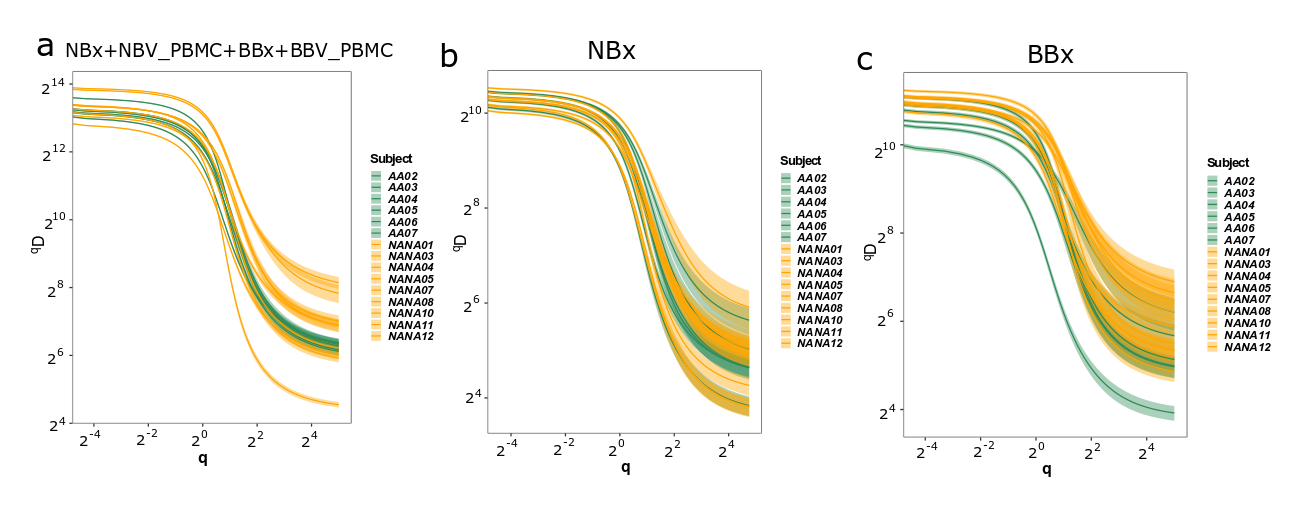


**Supplementary Fig. 3** Clonal diversity analysis using the generalized diversity index proposed by Hill (Hill, 1973) with uniform re-sampling to correct for sequencing depth, performed by Alakazam v0.2.5 (Stern et al., 2014;Gupta et al., 2015). The diversity index (^q^D) was calculated over a range of diversity orders (q) and plotted for each individual as a smooth curve for a) all biopsy samples combined, b) nasal biopsies only and c) bronchial biopsies only.  While the combined and nasal samples from the asthmatics and the non-atopic controls show similar diversity, sequences from the bronchial mucosa from the asthmatics are less diverse both in terms of both the numbers of unique sequences (smallest q) and sizes of the dominant clone (largest q), compared to those from the non-atopic controls.


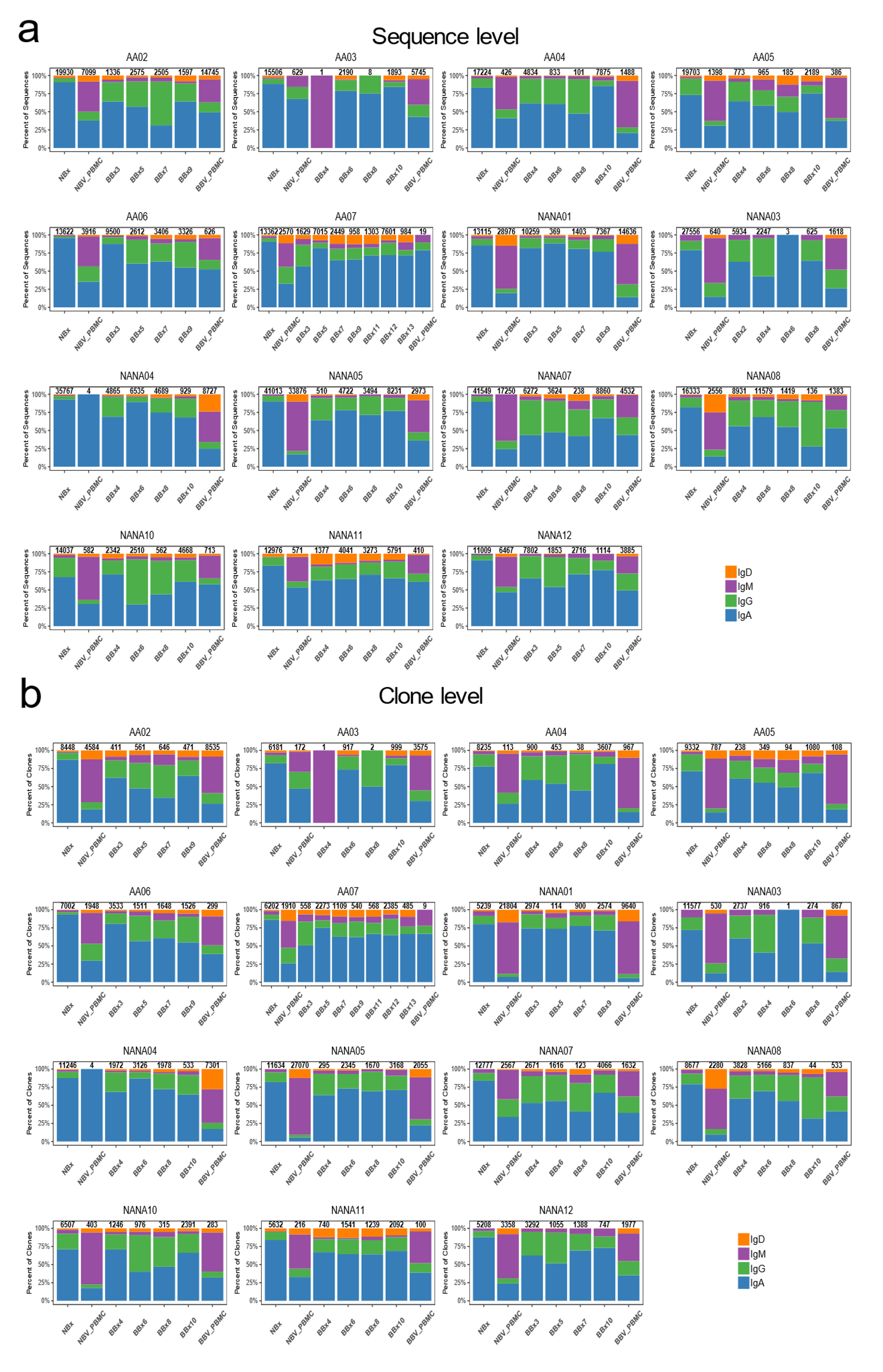


**Supplementary Fig. 4** Predominance of IgA, IgG and IgM clones from nasal and bronchial biopsies and the peripheral blood respectively. The figure shows isotype frequencies in nasal biopsies (NBx), bronchial biopsies (BBx) and contemporaneous peripheral blood samples (NBV_PBMC and BBV_PBMC, respectively) expressed as percentages of the total numbers of **a)** sequences and **b)** clones of isotype IgD (orange), IgM (purple), IgG (green) and IgA (blue), (the total numbers of sequence and clones from a given sample are listed above the bar). A few samples with very few sequences (BBx4 and BBx8 in AA03, BBx6 in NANA03) deviate from the common pattern. The isotype distribution is fairly similar in the asthmatics and non-atopic controls except that IgD clones tend to be more frequent in the bronchial biopsy samples from the asthmatics compared to the non-atopic controls. For each subject, between sample variability may reflect variable sequence numbers and coverage and, in the case of the biopsies, possible variability of cellular composition, for example the possible presence of germinal centre-like structures previously identified in bronchial biopsies (Pillai et al., 2016).


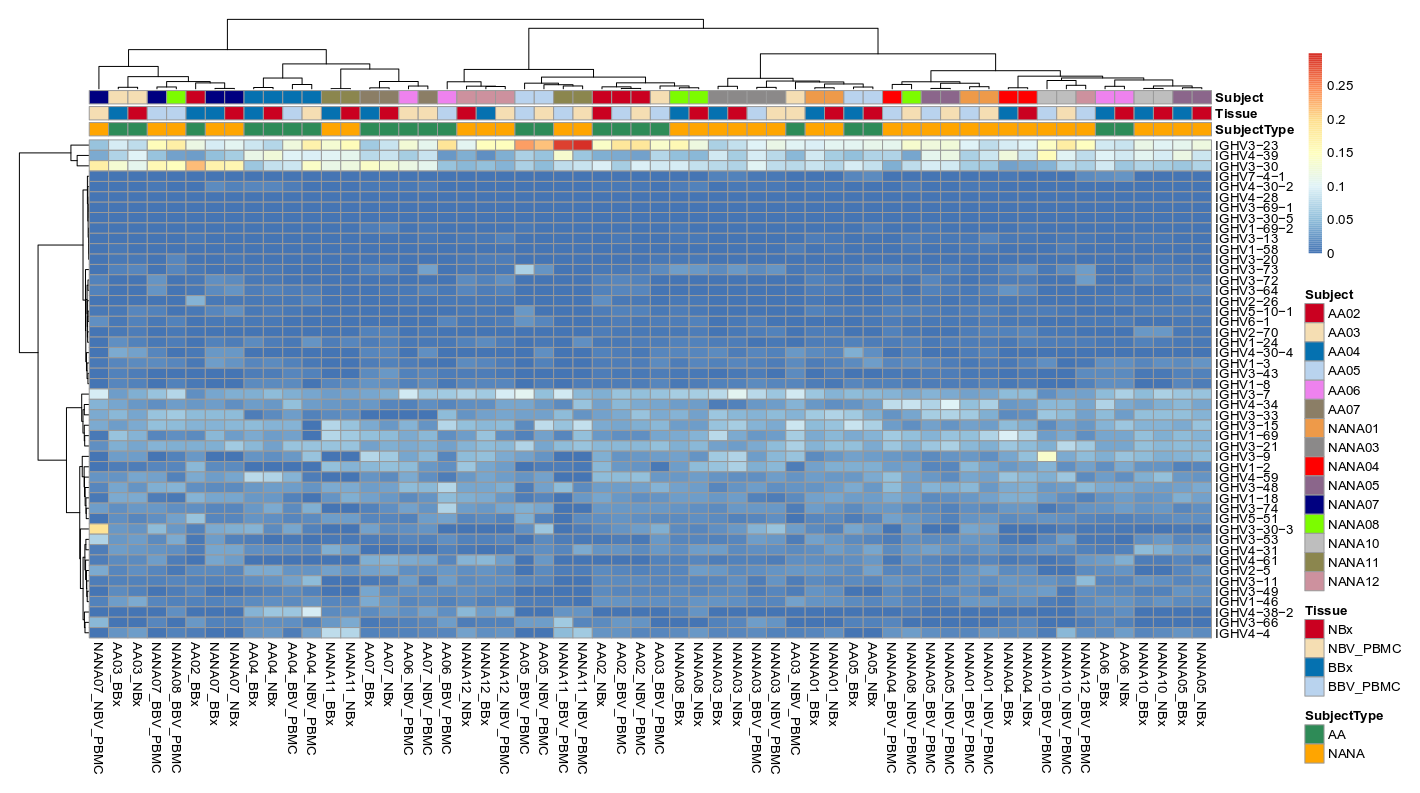


**Supplementary Fig. 5** Subject specific IGHV-gene profiles do not differ between atopic asthmatics (AA) and non-atopic, non-asthmatic controls (NANA). Heatmap showing IGHV-gene usage in the nasal (NBx) and bronchial (BBx) mucosa biopsies and contemporaneous peripheral blood samples (NBV_PBMC and BBV_PBMC, respectively) from each individual. IGHV3-23, IGHV3-30 and IGHV4-39 are the most commonly used IGHV genes in all samples. The four samples from each individual tend to cluster, suggesting that each individual has a unique IGVH-gene usage pattern.


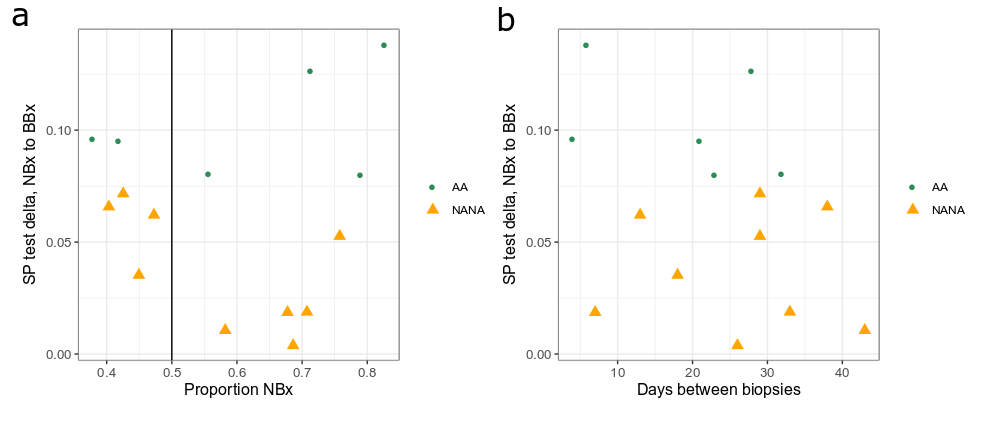


**Supplementary Fig. 6** Lack of correlation between the enrichment of migration events from the nasal to the bronchial tissue and both **a)** the proportion of nasal mucosal sequences and **b)** the numbers of days between the sampling of the nasal and bronchial biopsies in both atopic asthmatics (green circles) and non-atopic controls (yellow triangles), suggesting that the finding of enrichment of migration from the nasal to the bronchial tissue is not biased by the unavoidable variability in the biopsy size or by the sampling protocol allowing up to two months between study visits.


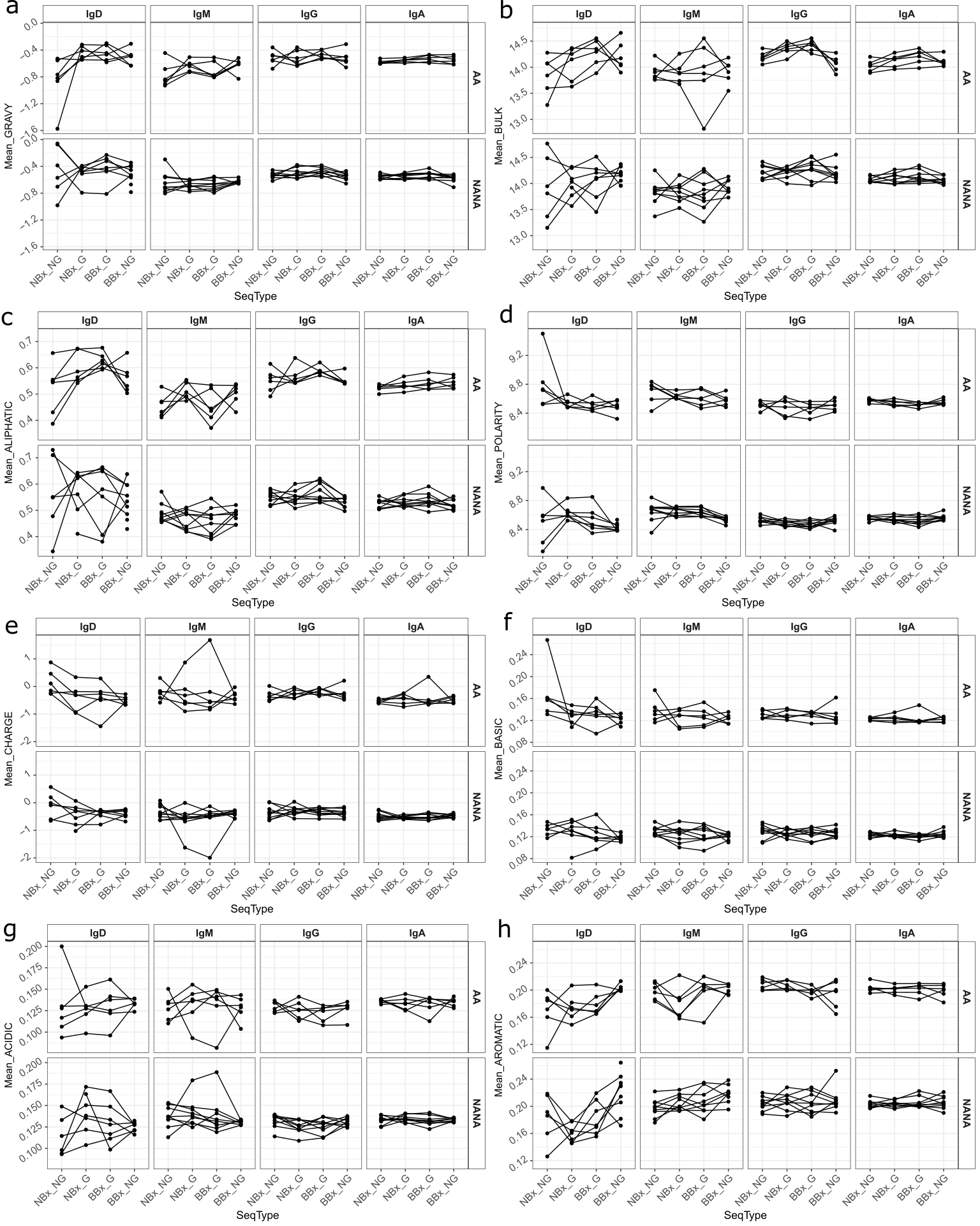


**Supplementary Fig. 7** Lack of significant differences in amino acid compositions of the complementarity determining region 3 (CDR3) sequences from globally and non-globally expressed antibodies in both the asthmatics and the non-atopic controls. Eight different properties of the CDR3 amino acids were compared using Alakazam (Gupta et al., 2015) for sequences from the four antibody isotypes (IgD, IgM, IgG and IgA) from clones found only in the nasal mucosa (NBx_NG), only the bronchial mucosa (BBx_NG) or at both sites (NBx_G, BBx_G).. Samples from the atopic asthmatic (AA) and non-atopic, non-asthmatic (NANA) control samples were analyzed separately. The analyzed CDR3 amino acid properties included the mean **a)** GRAVY index (the grand average of hydropathicity), **b)** bulk, **c)** aliphatic contents, **d)** polarity, **e)** charge, **f)** basic contents, **g)** acidic contents and **h)** aromatic contents of the CDR3s.


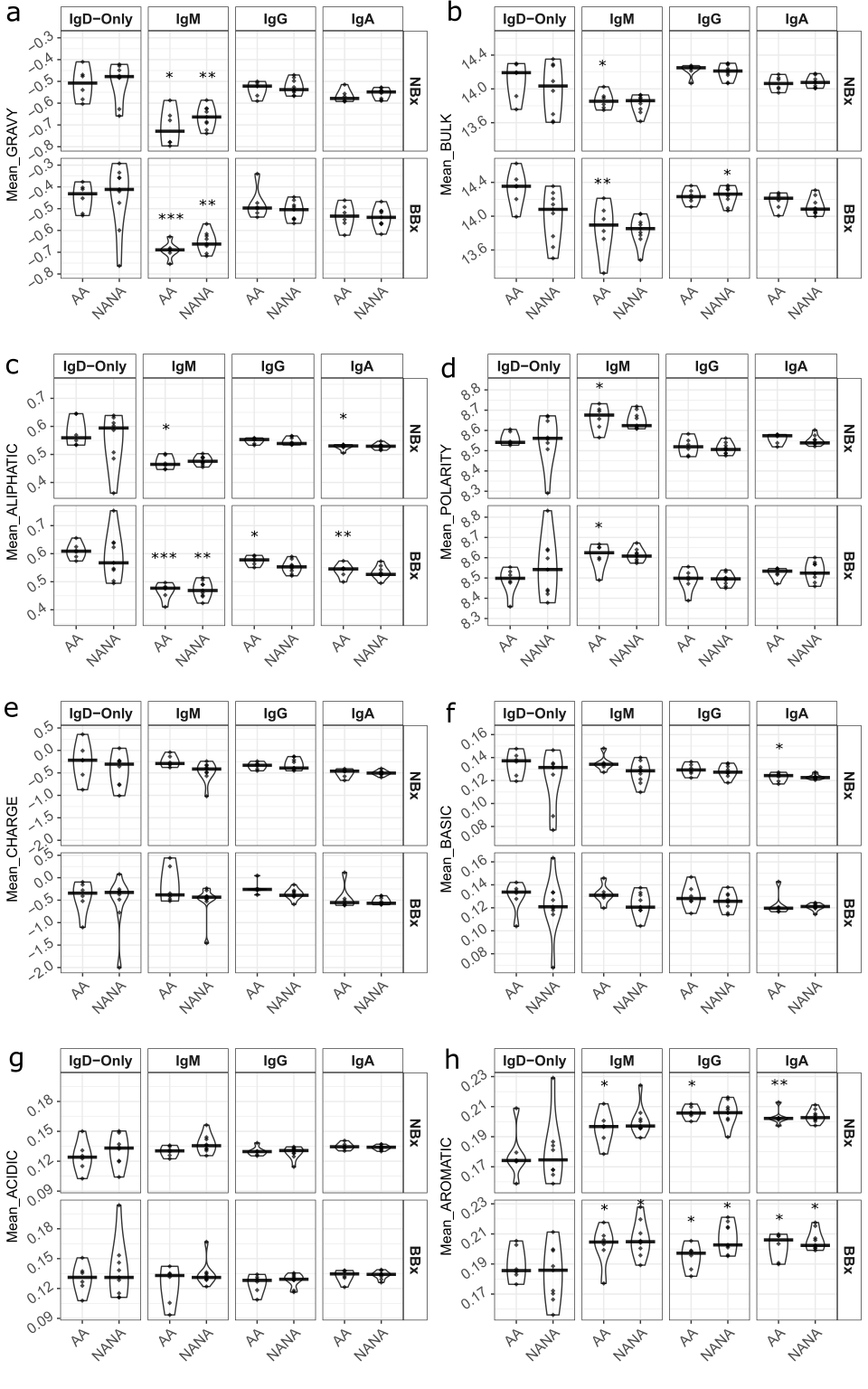


**Supplementary Fig. 8** Unique features of the properties of the amino acids forming the complementarity determining region 3 (CDR3) regions of the IgD-only, compared with the IgM, IgG and IgA clonal sequences from the nasal and bronchial biopsies evaluated using Alakazam (Gupta et al., 2015). Samples from the atopic asthmatics (AA) and non-atopic, non-asthmatics (NANA) were analyzed separately. The amino acid properties were the mean **a)** GRAVY index (the grand average of hydropathicity), **b)** bulk, **c)** aliphatic contents, **d)** polarity, **e)** charge, **f)** basic contents, **g)** acidic contents and **h)** aromatic contents. Statistically significant differences between the IgD-only sequences and other sequences are indicated on the figure * = p<0.05, ** = p<0.01 and ***=p<0.001. These data suggest that the IgD-only sequences are selected not only for long CDR3 regions with over-use of the IGHJ6 gene segment (see Fig. 8c and 8d), but also for certain amino acid properties.


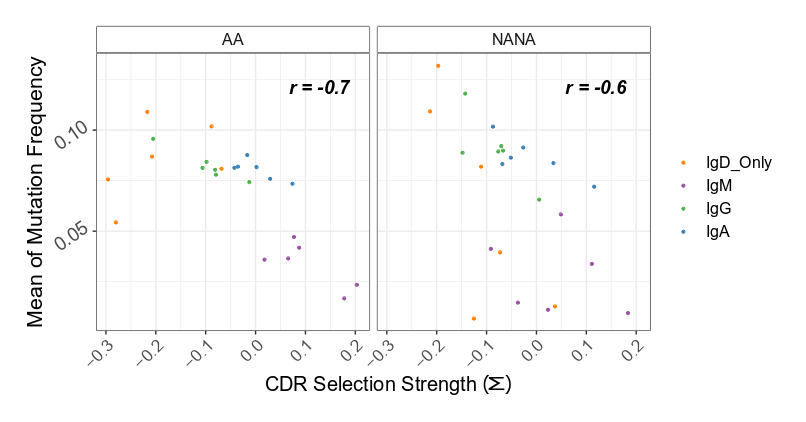


**Supplementary Fig. 9** Negative correlation between the CDR selection strength (∑) as calculated using BASELINe and the mean mutation frequency for sequences from IgD-only cells and cells of each isotype, IgM, IgA and IgG, from both asthmatics (AA) and non-atopic, non-asthmatics controls (NANA). Each dot represents data combined from all bronchial biopsy samples from one individual.

## Supplementary Tables

| Identifier | Sex | Age | Allergies* | Asthma Medication | Rhinitis** |
| --- | --- | --- | --- | --- | --- |
| Atopic Asthmatics | | | | | |
| AA02 | M | 35 | HDM, dog | Seretide (1000 mcg/day) | No |
| AA03 | F | 38 | Early flowering trees, HDM, cat, dog | Symbicort 200/6 (800 mcg/day) | Yes |
| AA04 | M | 40 | Grass, early and mid flowering trees, HDM, cat | Flutiform 125 (250 mg/day **fluticasone propionate** and 10 mcg/day formoterol) | Yes |
| AA05 | F | 50 | Grass, HDM, cat, dog | Flutiform 250 (1000 mg/day **fluticasone propionate** and 40 mcg/day formoterol) | Yes |
| AA06 | F | 23 | Dog, aspergillus | 200 mcg/day inhaled steroid (brand unknown) | Yes |
| AA07 | M | 21 | Grass, early and mid-flowering trees, cat, dog, Alternaria, Cladosporium | Clenil Modulite (200 mcg/day) | Yes |
| Non-Atopic, Non-Asthmatic controls | | | | | |
| NANA01 | M | 34 | N/A | N/A | No |
| NANA03 | F | 32 | N/A | N/A | No |
| NANA04 | F | 36 | N/A | N/A | No |
| NANA05 | F | 23 | N/A | N/A | No |
| NANA07 | M | 25 | N/A | N/A | No |
| NANA08 | F | 31 | N/A | N/A | No |
| NANA10 | M | 23 | N/A | N/A | No |
| NANA11 | M | 51 | N/A | N/A | No |
| NANA12 | F | 26 | N/A | N/A | No |

* As identified by a positive skin prick test. The skin prick tests contained allergens from the following organisms;

HDM: Dermatophagoides pteronyssinus

Grass pollen mixture: Yorkshire Fog, Cocksfoot, Rye Grass, Timothy Grass, Meadow Grass and

Tall fescue

Tree pollen mixtures; *Early flowering trees:* Alder, Hazel, Poplar, Elm, Willow and *Mid flowering trees:* Birch, beech oak, plane

** Self-reported symptoms of rhinitis

**Supplementary Table 1** Clinical data for study subjects

| From | To | Mean 𝛿 | p-value |
| --- | --- | --- | --- |
| Left | NBx | -0.026 | **< 0.01** |
| Left | Right | -0.018 | **< 0.01** |
| NBx | Left | 0.04 | **< 0.01** |
| NBx | Right | 0.042 | **< 0.01** |
| Right | Left | -0.012 | **0.01** |
| Right | NBx | -0.026 | **< 0.01** |

**Supplemental Table 2** Phylogenetic analysis of B cell trafficking patterns between the nasal mucosa (NBx), the bronchial mucosa of the left lung (Left) and right lungs (Right) in individual AA07. Only sequences from these tissues were included in this analysis. For each comparison, the proportion of predicted tissue changes that occurred in the specified direction along B cell lineage trees was compared to the proportion of these changes observed in trees within randomized tip locations. Mean 𝛿 is the mean difference in the proportion of trafficking events in the specified direction in observed versus randomized lineage trees. Mean 𝛿 > 0 indicates a greater proportion of trafficking events in the specified direction in observed trees, while mean 𝛿 < 0 indicates the opposite. The p-value indicates the significance of this difference. If mean 𝛿 > 0, the reported p-value is the proportion of 100 bootstrap/permutation replicates in which 𝛿 ≤ 0. If mean 𝛿 < 0, the reported p-value is the proportion of 100 bootstrap/permutation replicates in which 𝛿 ≥ 0. p-values < 0.025 are displayed in bold. Numbers are reported to two significant digits.

| Subject | Number of shared clones | More mutation in bronchial fraction,  *p-value** |
| --- | --- | --- |
| **AA02** | 634 | **0.000** |
| **AA03** | 915 | **0.014** |
| **AA04** | 1655 | **0.007** |
| **AA05** | 742 | **0.020** |
| **AA06** | 1865 | **0.000** |
| **AA07** | 1396 | **0.000** |
| **NANA01** | 1528 | **0.000** |
| **NANA03** | 1593 | **0.037** |
| **NANA04** | 2884 | 0.354 |
| **NANA05** | 2350 | **0.000** |
| **NANA07** | 2754 | 0.226 |
| **NANA08** | 2729 | 0.161 |
| **NANA10** | 1262 | 0.291 |
| **NANA11** | 1423 | **0.014** |
| **NANA12** | 1189 | 0.973 |

* statistically significant values are in bold

**Supplementary Table 3** Mutation frequency in the nasal versus bronchial clone members of clones spanning both tissues.

| Subject | Mean 𝛿 | p-value |
| --- | --- | --- |
| AA02 | 0.13 | **< 0.01** |
| AA03 | 0.08 | **< 0.01** |
| AA04 | 0.08 | **< 0.01** |
| AA05 | 0.14 | **< 0.01** |
| AA06 | 0.095 | **< 0.01** |
| AA07 | 0.096 | **< 0.01** |
| NANA01 | 0.066 | **< 0.01** |
| NANA03 | 0.053 | **< 0.01** |
| NANA04 | 0.019 | **< 0.01** |
| NANA05 | 0.019 | **0.01** |
| NANA07 | 0.0038 | 0.29 |
| NANA08 | 0.072 | **< 0.01** |
| NANA10 | 0.011 | 0.26 |
| NANA11 | 0.062 | **< 0.01** |
| NANA12 | 0.035 | **< 0.01** |

**Supplementary Table 4** Phylogenetic analysis of B cell trafficking patterns between the nasal and bronchial mucosa. Only sequences from nasal and bronchial biopsies were included in this analysis. For each individual, the proportion of predicted tissue changes that occurred from the nasal mucosa to the bronchial mucosa along B cell lineage trees was compared to the proportion of these changes observed in trees within randomized tip locations. Mean 𝛿 is the mean difference in the proportion of trafficking events from the nasal to the bronchial mucosa in observed vs. randomized lineage trees. Mean 𝛿 > 0 indicates a greater proportion of trafficking events from the nasal to the bronchial mucosa in observed trees. The reported p-value that 𝛿 > 0 is the proportion of 100 bootstrap/permutation replicates in which 𝛿 ≤ 0. p < 0.05 are displayed in **bold**. Numbers are reported to two significant digits.

|  |  |  |  | **To** |  |
| --- | --- | --- | --- | --- | --- |
| **Subject** | **From** | **NBx** | **BBx** | **NBV_PBMC** | **BBV_PBMC** |
| AA02 | NBx | - | **< 0.02** | **0.04** | **< 0.02** |
|  | BBx | 1 | - | 1 | 1 |
|  | NBV_PBMC | 1 | 0.22 | - | 1 |
|  | BBV_PBMC | 1 | 0.24 | 0.98 | - |
| AA03 | NBx | - | **< 0.02** | 1 | 0.8 |
|  | BBx | 1 | - | 0.72 | 0.2 |
|  | NBV_PBMC | 1 | 0.54 | - | 0.98 |
|  | BBV_PBMC | 1 | 0.48 | 0.46 | - |
| AA04 | NBx | - | **< 0.02** | 0.22 | 0.74 |
|  | BBx | 1 | - | 0.98 | 0.96 |
|  | NBV_PBMC | 1 | 0.98 | - | 0.52 |
|  | BBV_PBMC | 1 | 0.7 | 0.82 | - |
| AA05 | NBx | - | **< 0.02** | 0.14 | 0.1 |
|  | BBx | 1 | - | 0.82 | 0.62 |
|  | NBV_PBMC | 0.48 | 0.34 | - | 0.56 |
|  | BBV_PBMC | 1 | 0.94 | 0.98 | - |
| AA06 | NBx | - | **< 0.02** | 1 | **< 0.02** |
|  | BBx | 1 | - | 0.4 | **< 0.02** |
|  | NBV_PBMC | 1 | 0.68 | - | 1 |
|  | BBV_PBMC | 0.9 | 0.94 | 1 | - |
| AA07 | NBx | - | **< 0.02** | 0.62 | - |
|  | BBx | 1 | - | 0.34 | - |
|  | NBV_PBMC | 1 | 0.96 | - | - |
| NANA01 | NBx | - | **< 0.02** | 0.3 | 0.66 |
|  | BBx | 0.92 | - | 0.86 | 0.98 |
|  | NBV_PBMC | 0.98 | 1 | - | 1 |
|  | BBV_PBMC | 1 | 0.98 | 0.98 | - |
| NANA03 | NBx | - | **< 0.02** | 0.64 | 0.42 |
|  | BBx | 1 | - | 0.46 | 0.1 |
|  | NBV_PBMC | 0.88 | 0.38 | - | 0.34 |
|  | BBV_PBMC | 1 | 1 | 0.12 | - |
| NANA04 | NBx | - | **< 0.02** | **0.04** | 1 |
|  | BBx | 0.92 | - | 0.96 | 0.58 |
|  | NBV_PBMC | 1 | 1 | - | 1 |
|  | BBV_PBMC | 1 | 0.98 | 1 | - |
| NANA05 | NBx | - | **< 0.02** | 1 | 1 |
|  | BBx | 0.6 | - | 0.08 | 0.22 |
|  | NBV_PBMC | 0.96 | 1 | - | 0.44 |
|  | BBV_PBMC | 1 | 0.94 | 1 | - |
| NANA07 | NBx | - | **< 0.02** | 0.82 | 1 |
|  | BBx | 0.28 | - | 0.34 | 0.62 |
|  | NBV_PBMC | 0.1 | 0.9 | - | 1 |
|  | BBV_PBMC | 1 | 1 | 1 | - |
| NANA08 | NBx | - | **< 0.02** | 0.66 | 0.86 |
|  | BBx | 1 | - | 0.94 | 0.96 |
|  | NBV_PBMC | 1 | 0.94 | - | 1 |
|  | BBV_PBMC | 1 | 1 | 1 | - |
| NANA10 | NBx | - | 0.24 | 0.56 | **0.02** |
|  | BBx | 0.64 | - | 0.24 | 0.46 |
|  | NBV_PBMC | 0.24 | 0.3 | - | **0.02** |
|  | BBV_PBMC | 1 | 1 | 0.54 | - |
| NANA11 | NBx | - | **< 0.02** | 0.88 | **< 0.02** |
|  | BBx | 1 | - | 1 | 0.18 |
|  | NBV_PBMC | 0.84 | 0.72 | - | 0.94 |
|  | BBV_PBMC | 1 | 1 | **< 0.02** | - |
| NANA12 | NBx | - | **< 0.02** | 0.1 | 0.6 |
|  | BBx | 0.06 | - | 0.98 | 1 |
|  | NBV_PBMC | 1 | 1 | - | 1 |
|  | BBV_PBMC | 0.62 | 0.7 | 1 | - |

**Supplementary Table 5** Phylogenetic analysis of B cell trafficking patterns between the nasal mucosa (NBx), bronchial mucosa (BBx), peripheral blood drawn at the time of the nasal biopsies (NBV_PBMC) and peripheral blood taken at the time of the bronchoscopy (BBV_PBMC) in each of the 15 individual study subjects (AA = atopic asthmatics and NANA = non-atopic, non=asthmatics). Cells show the p-value that 𝛿 > 0 between the two tissues in the direction specified (see description of statistical test in **Supplementary Tables 2** and **4**). A p < 0.05 indicates a significantly greater proportion of trafficking events occurred in the specified direction in observed B cell lineage trees compared to randomized trees. p < 0.05 are displayed in **bold**. Numbers are reported to two significant digits.

# References

Alamyar, E., Duroux, P., Lefranc, M.P., and Giudicelli, V. (2012). IMGT(®) tools for the nucleotide analysis of immunoglobulin (IG) and T cell receptor (TR) V-(D)-J repertoires, polymorphisms, and IG mutations: IMGT/V-QUEST and IMGT/HighV-QUEST for NGS. *Methods Mol Biol* 882**,** 569-604.

Csardi, G.T., Nepusz (2006). The igraph software package for complex network research. *InterJournal Complex Systems* 1695.

Felsenstein, J. (1985). CONFIDENCE LIMITS ON PHYLOGENIES: AN APPROACH USING THE BOOTSTRAP. *Evolution* 39**,** 783-791.

Felsenstein, J. (1989). PHYLIP - phylogeny inference package (version 3.2). *Cladistics* 5**,** 164-166.

Gupta, N.T., Vander Heiden, J.A., Uduman, M., Gadala-Maria, D., Yaari, G., and Kleinstein, S.H. (2015). Change-O: a toolkit for analyzing large-scale B cell immunoglobulin repertoire sequencing data. *Bioinformatics* 31**,** 3356-3358.

Hill, M. (1973). Diversity and evenness: a unifying notation and its consequences. *Ecology* 54**,** 427-432.

Hoehn, K.B., Pybus, O.G., and Kleinstein, S.H. (2020). Phylogenetic analysis of migration, differentiation, and class switching in B cells. *bioRxiv***,** 2020.2005.2030.124446.

Hsieh, T.C., Ma, K.H., and Chao, A. (2016). iNEXT: an R package for rarefaction and extrapolation of species diversity (Hill numbers). *Methods in Ecology and Evolution* 7**,** 1451-1456.

Pillai, P., Chan, Y.C., Wu, S.Y., Ohm-Laursen, L., Thomas, C., Durham, S.R., Menzies-Gow, A., Rajakulasingam, R.K., Ying, S., Gould, H.J., and Corrigan, C.J. (2016). Omalizumab reduces bronchial mucosal IgE and improves lung function in non-atopic asthma. *Eur Respir J* 48**,** 1593-1601.

Soto, C., Bombardi, R.G., Branchizio, A., Kose, N., Matta, P., Sevy, A.M., Sinkovits, R.S., Gilchuk, P., Finn, J.A., and Crowe, J.E., Jr. (2019). High frequency of shared clonotypes in human B cell receptor repertoires. *Nature* 566**,** 398-402.

Stern, J.N., Yaari, G., Vander Heiden, J.A., Church, G., Donahue, W.F., Hintzen, R.Q., Huttner, A.J., Laman, J.D., Nagra, R.M., Nylander, A., Pitt, D., Ramanan, S., Siddiqui, B.A., Vigneault, F., Kleinstein, S.H., Hafler, D.A., and O'connor, K.C. (2014). B cells populating the multiple sclerosis brain mature in the draining cervical lymph nodes. *Sci Transl Med* 6**,** 248ra107.

Uduman, M., Yaari, G., Hershberg, U., Stern, J.A., Shlomchik, M.J., and Kleinstein, S.H. (2011). Detecting selection in immunoglobulin sequences. *Nucleic Acids Res* 39**,** W499-504.

Vander Heiden, J.A., Yaari, G., Uduman, M., Stern, J.N., O'connor, K.C., Hafler, D.A., Vigneault, F., and Kleinstein, S.H. (2014). pRESTO: a toolkit for processing high-throughput sequencing raw reads of lymphocyte receptor repertoires. *Bioinformatics* 30**,** 1930-1932.

Yaari, G., Uduman, M., and Kleinstein, S.H. (2012). Quantifying selection in high-throughput Immunoglobulin sequencing data sets. *Nucleic Acids Res* 40**,** e134.

Yaari, G., Vander Heiden, J.A., Uduman, M., Gadala-Maria, D., Gupta, N., Stern, J.N., O'connor, K.C., Hafler, D.A., Laserson, U., Vigneault, F., and Kleinstein, S.H. (2013). Models of somatic hypermutation targeting and substitution based on synonymous mutations from high-throughput immunoglobulin sequencing data. *Front Immunol* 4**,** 358.

Ye, J., Ma, N., Madden, T.L., and Ostell, J.M. (2013). IgBLAST: an immunoglobulin variable domain sequence analysis tool. *Nucleic Acids Res* 41**,** W34-40.
